# Task-adaptive multi-view learning with ordinal graph and continuous-score paths for public hospital performance stratification

**DOI:** 10.3389/fpubh.2026.1890759

**Published:** 2026-10-30

**Authors:** Yajie Gan, Dezhi Li

**Affiliations:** Southwest Medical University Affiliated Hospital, Luzhou, Sichuan, China

**Keywords:** graph learning, health insurance, ordinal classification, patient safety, performance stratification, public hospital, value-based purchasing

## Abstract

**Introduction:**

Public reporting provides repeated measures of hospital quality, spending, safety, and patient experience. Outcomes are ordered, and feature availability can change across releases. We evaluated Health–Insurance–Operational–Proxy synergy-aware Ordinal Graph Learning (HIOP–GL) for next-release performance stratification among publicly owned US hospitals.

**Methods:**

Twelve Centers for Medicare & Medicaid Services (CMS) snapshots from 2023 to 2026 were aligned without concurrent targets. For the next-release overall hospital-rating task (PH-Star), development used 1,849 hospital-release observations from 341 hospitals, followed by 1,236 post-development observations from 338 hospitals. Because annual Hospital Value-Based Purchasing total performance scores (TPS) repeated within fiscal years, the corresponding PH-VBP task used FY2024 development (*n* = 321) and FY2025 tuning (*n* = 301) for selection. After architecture, loss, cutoff, and epoch choices were fixed using FY2024–FY2025 data, the model was refitted on both years (622 hospital-year records) and evaluated in FY2026 (*n* = 308). PH-Star used a three-relation ordinal graph path specified before evaluation, whereas PH-VBP used a graph bypass, continuous TPS head, and tier cutoffs fixed from FY2024.

**Results:**

Across five fits, PH-Star quadratic weighted kappa (QWK) was 0.816 ± 0.009 on the temporal test and 0.757 ± 0.013 after development. The two-year-refit PH-VBP model reached QWK 0.678 ± 0.021 in FY2026; ensemble QWK was 0.717 (95% CI 0.657–0.773). Hospital-disjoint and Census-region-disjoint cross-fitting yielded QWK 0.689 and 0.636. A PH-Star ensemble held constant after development achieved QWK 0.637 (95% CI 0.546–0.718; *n* = 229) in zero-overlap California non-public hospitals. Independent-authority checks found concordance with 2023 California multi-measure ratings (*n* = 243, ρ = 0.247, 95% CI 0.125–0.366; Holm *p* = 0.0006) and 2024 New York all-payer mortality composites in 57 exact-linked hospitals (ρ = 0.451, 95% CI 0.195–0.663; Holm *p* = 0.0010). Concordance varied by criterion, with no supported California mortality association.

**Discussion:**

HIOP-GL's clearest advantage was ordinal agreement in later-release evaluation. Macro-F1 and low-tier recall remained metric-dependent, and hospital-grouped PH-Star QWK was 0.661 vs. 0.730 for ordinal logistic. Ownership transport and multi-state independent-criterion concordance extend the assessment beyond development. The results support outcome-specific modeling for retrospective ordinal stratification; prospective same-outcome validation across independent systems remains necessary.

## 1 Introduction

Publicly accountable hospitals are assessed across clinical outcomes, patient experience, timeliness, safety, and expenditure. These domains are related but not interchangeable. An institution may have favorable experience scores and comparatively high episode spending, or acceptable overall ratings and persistent infection-control concerns. The Donabedian structure–process–outcome framework and value-based care both motivate multidimensional assessment rather than reliance on a single indicator (1, 2). Measures used for public accountability must also be interpretable and sufficiently reliable for quality-improvement use (3). Evidence from public report cards, national patient-experience reporting, and Medicare hospital pay-for-performance indicates that reported measures and incentive effects require cautious interpretation (4–6).

Public hospital data pose a specific statistical problem. The outcomes such as overall hospital ratings and Hospital Value-Based Purchasing (HVBP) total performance scores are ordered, whereas predictors are high-dimensional, repeatedly released, and incompletely reported. Mixed-type tables with missing values also require explicit preprocessing (7). Hospitals additionally share geographic and organizational contexts. Conventional tabular models can represent non-linear associations, but they do not directly encode three features of this problem: order, repeated hospital observations, and multiple institution-level relations. Graph models can represent relational structure, but they may leak concurrent outcome information unless edge construction and release timing are explicitly controlled.

Previous studies caution that administrative algorithms can reproduce systematic disparities when their targets or inputs are poorly specified (8). Tree ensembles and tabular neural methods provide the main non-relational comparators (9–15). Ordinal regression and rank-consistent neural objectives model ordered labels, while graph convolution models related observations (16–19). Prediction-model guidance also emphasizes performance decomposition, calibration, transparent reporting, risk-of-bias assessment, and clearly defined validation populations (20–24). This study examines these requirements jointly under reproducible timing. The analysis covers ordered hospital outcomes, multi-domain missing predictors, distinct hospital relations, repeated releases, calibration, and transportability to hospitals not represented in model development.

The study centers on outcome-specific modeling for ordinal hospital stratification. Release-level PH-Star and fiscal-year-specific PH-VBP designs preserve the timing and update frequency of each outcome (Sections 2.1, 2.2). The shared multi-view encoder supports a three-relation ordinal graph path for PH-Star and a validation-selected continuous-TPS graph-bypass path for PH-VBP (Sections 2.4, 2.5). Four later CMS release pairs and FY2026 PH-VBP evaluation test temporal transport after model choices are held constant. Hospital-disjoint and Census-region-disjoint cross-fitting examine transfer across the corresponding groups, with hospital-block uncertainty and explicit schema and target-drift assessment (Sections 2.7 and 3). Same-outcome ownership transport and concordance with independently maintained state criteria provide complementary assessments beyond the development cohort. Component ablations, calibration, subgroup results, and attribution analyses clarify which parts of the framework support ordinal agreement and how that agreement relates to exact-tier classification. The overall study workflow and task-specific model architecture are summarized in Figures 1 and 2, respectively.

**Figure 1:**
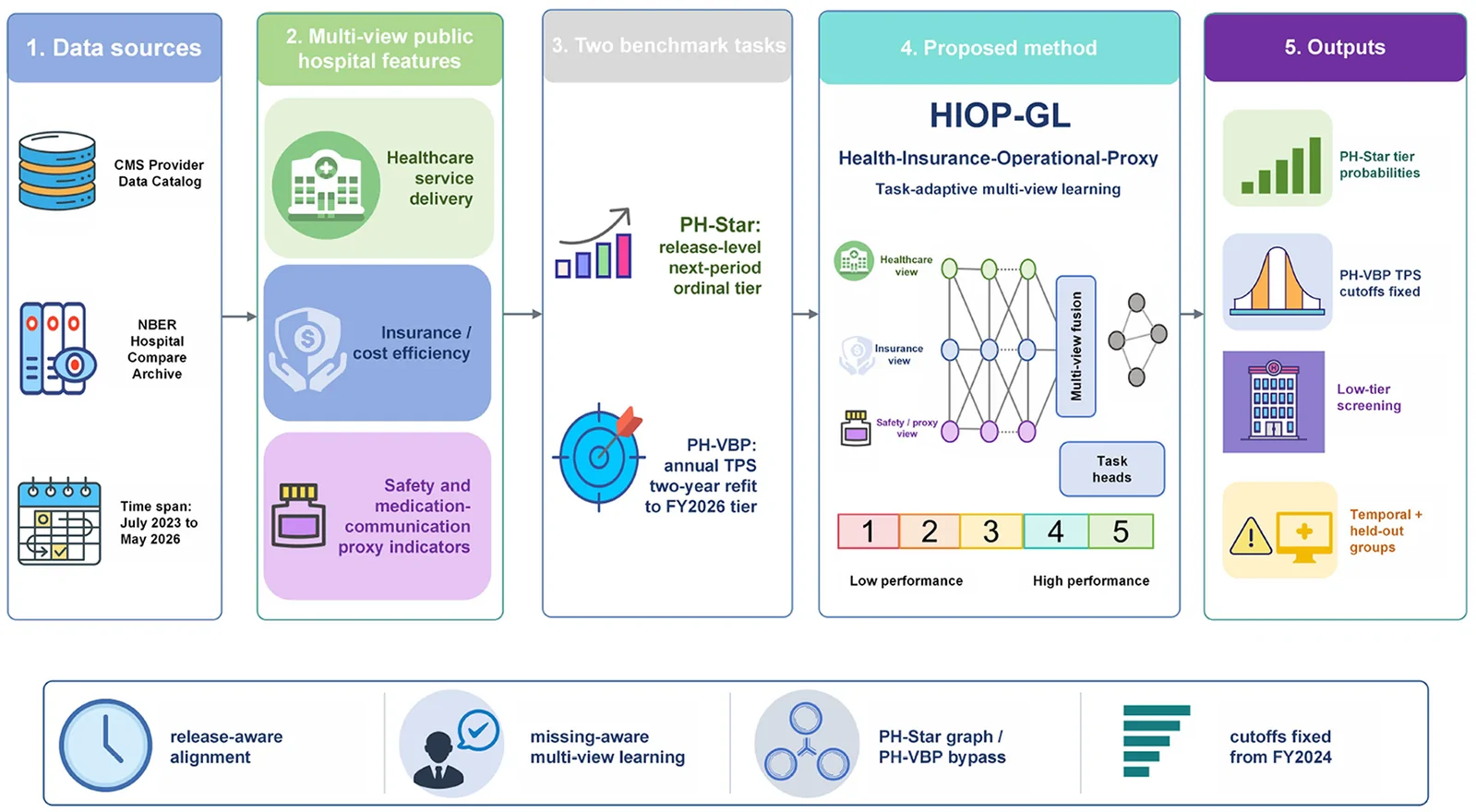
Study workflow. Consecutive public releases are aligned so that predictors at release *t* estimate outcomes at release *t*+1. Continuous measures are separated into healthcare-service, insurance/cost, and safety/medication-communication proxy views. PH-Star uses three target-independent hospital relations and an ordinal head. PH-VBP uses FY2024 development and FY2025 tuning to select its graph-bypass continuous-TPS configuration. That configuration is held constant during the 2-year historical refit, and FY2026 predictions are mapped through cutoffs fixed from FY2024. Hospital- and Census-region-disjoint temporal cross-fitting provide separated-group transport analyses.

**Figure 2:**
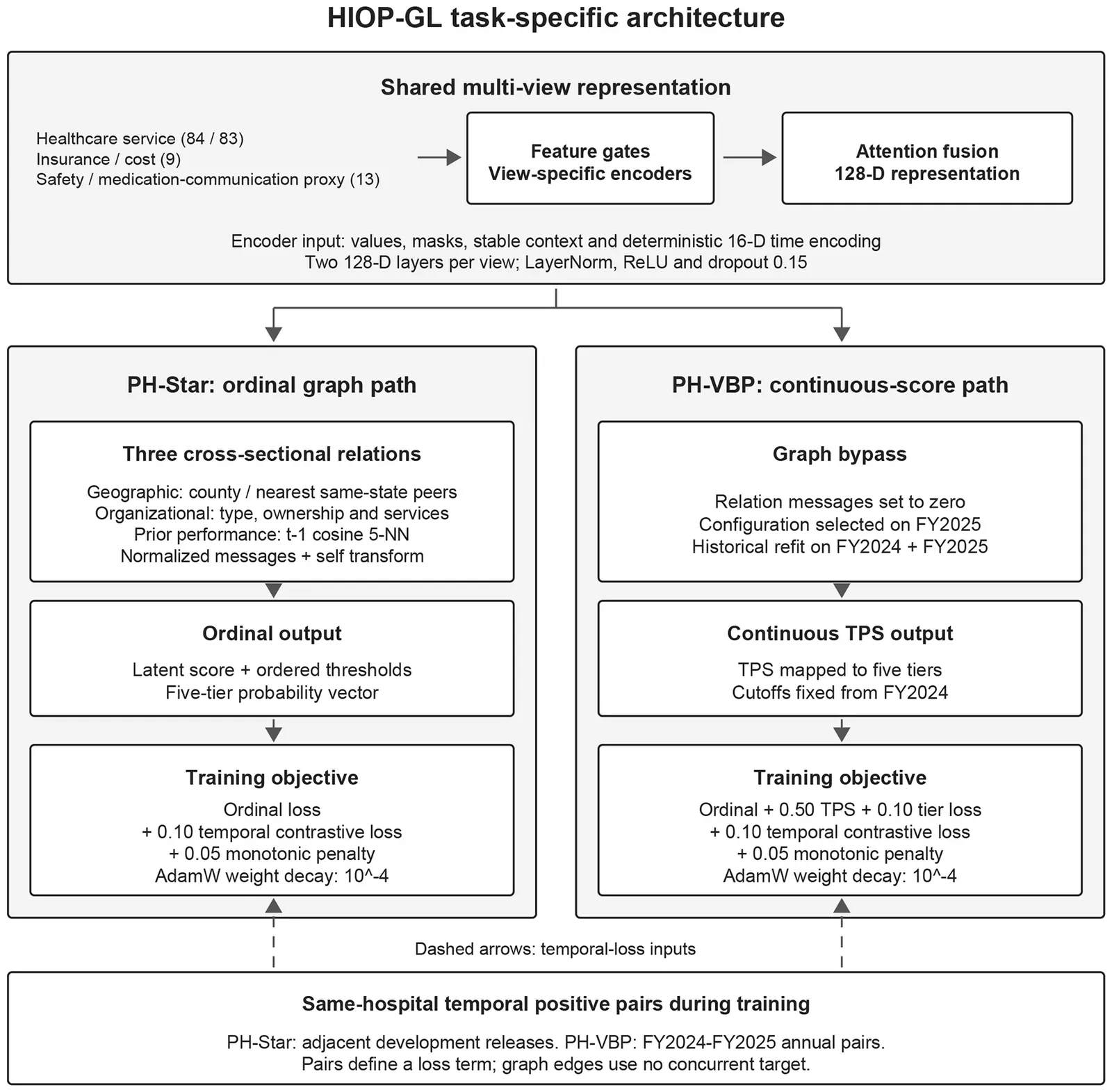
Implemented task-specific HIOP framework. Shared feature gates, view-specific encoders, and attention fusion feed two outcome-specific paths. Healthcare-view counts are shown as PH-Star / PH-VBP. PH-Star uses three cross-sectional adjacency matrices and a cumulative ordinal head. PH-VBP bypasses graph messages, predicts continuous TPS, and applies cutoffs fixed from FY2024. Both training objectives retain ordinal and monotonic terms; PH-VBP additionally uses TPS and tier losses. Dashed arrows connect same-hospital temporal pairs directly to the training objectives: adjacent development releases for PH-Star and FY2024–FY2025 annual pairs for the 2-year PH-VBP refit. These pairs are not a fourth graph relation.

## 2 Materials and methods

### 2.1 Data sources, releases, and cohort

We downloaded 12 dated Hospital Compare snapshots from the CMS Provider Data Catalog: 6 July 2023, 6 October 2023, 31 January 2024, 24 April 2024, 31 July 2024, 30 October 2024, 19 February 2025, 30 April 2025, 14 August 2025, 26 November 2025, 25 February 2026, and 13 May 2026 (25). Source tables covered hospital characteristics, HCAHPS patient experience, timely and effective care, complications and deaths, unplanned hospital visits, healthcare-associated infections, Medicare spending per beneficiary, payment measures, and HVBP TPS. Each predictor release was linked to the immediately following outcome release. No concurrent target entered predictor construction.

Public ownership was identified before outcome filtering by the prespecified case-insensitive pattern government| veterans health administration| department of defense| tribal. An eligible observation required a valid facility identifier, at least one retained predictor, and non-missing next-release PH-Star and PH-VBP outcomes. Predictor missingness was retained. Duplicate facility-measure rows were resolved by source reporting period and release-table order before wide-format alignment.

PH-Star used the original release-level panel. Training comprised 945 observations from feature releases October 2023, January 2024, and April 2024; validation comprised 301 observations from July 2024; and the prespecified temporal test comprised 603 observations from October 2024 and February 2025. Four later pairs, namely, April–August 2025, August–November 2025, November 2025–February 2026, and February–May 2026, contributed 300, 301, 308, and 327 outcome-complete observations, respectively. Their combined post-development set contained 1,236 observations from 338 hospitals.

PH-VBP required a fiscal-year-specific design. In the release-level panel, 624 hospital–fiscal-year groups contained multiple rows, and every such group repeated an identical TPS. We therefore used one pair per fiscal year: October 2023–January 2024 for FY2024 development (*n* = 321), October 2024–February 2025 for FY2025 tuning (*n* = 301), and November 2025–February 2026 for FY2026 temporal validation (*n* = 308). No PH-VBP hospital contributed more than one row within a fiscal year. Architecture, loss weights, cutoffs, and seed-specific epoch counts were selected using FY2024–FY2025 only. In this study, model locking means that these choices were fixed in advance before any FY2026 analysis. The final PH-VBP models were then refitted without outcome-based early stopping on the combined FY2024 and FY2025 records (622 hospital-year observations from 338 hospitals) and evaluated in FY2026. FY2026 outcomes were never used in preprocessing, fitting, cutoff estimation, fold assignment, or model selection. The task-specific temporal cohorts are summarized in Table 1.

**Table 1:** Task-specific temporal cohorts.

Task	Phase	Feature–outcome release(s)	Observations	Hospitals	Tiers 1–5	Low tier
PH-Star	Train	2023-10 through 2024-04 pairs	945	325	109/263/304/173/96	39.4%
	Validation	2024-07–2024-10	301	301	36/90/86/65/24	41.9%
	Temporal test	2024-10–2025-02; 2025-02–2025-04	603	302	72/176/173/134/48	41.1%
	Post-development	Four pairs ending 2025-08 through 2026-05	1,236	338	140/343/400/268/85	39.1%
PH-VBP	FY2024 development	2023-10–2024-01	321	321	64/65/62/65/65	40.2%
	FY2025 tuning	2024-10–2025-02	301	301	59/47/71/54/70	35.2%
	2-year historical refit	FY2024 + FY2025 pairs	622	338	123/112/133/119/135	37.8%
	FY2026 temporal validation	2025-11–2026-02	308	308	6/32/48/74/148	12.3%

Tier counts are ordered from 1 to 5. PH-Star counts are hospital-release observations; PH-VBP counts are one hospital observation per fiscal year.

External evaluation used the 2023 California Hospital Performance Ratings, 2024 California Hospital Inpatient Mortality Rates and Quality Ratings, and the official Licensed and Certified Healthcare Facility Crosswalk maintained by the California Department of Health Care Access and Information (HCAI) (26–28). We also used the 2024 hospital-level Inpatient Quality Indicator (IQI) composite dataset maintained by the New York State Department of Health (NYSDOH). This dataset applies Agency for Healthcare Research and Quality methods to all-payer SPARCS discharges (29). These state sources were not used for feature engineering, model fitting, architecture selection, epoch selection, threshold selection, or calibration.

### 2.2 Outcome definitions

PH-Star was the next-release CMS overall hospital rating, restricted to reported integer values 1 through 5. The reported rating was used directly as the ordered tier; 1 was lowest and 5 highest.

PH-VBP was the next-release HVBP TPS. Four cutoffs were estimated once from FY2024 development scores at empirical quintiles: 12.75, 17.50, 22.9167, and 29.50. These cutoffs were held constant for FY2025 tuning and FY2026 temporal validation. For score *s* and cutoffs *c*_1_, …, *c*_4_, the tier was defined in Equation 1 as


$$\begin{array}{l} y=1+\overset{4}{\underset{k=1}{\sum }}I\left(s>c_{k}\right). \end{array}$$ (1)

A score exactly equal to a cutoff was assigned to the lower tier. There were 2, 2, 1, and 2 exact FY2024 ties at the four boundaries. Mean TPS was 21.56 in FY2024, 22.51 in FY2025, and 29.84 in FY2026. Higher tiers indicated better reported performance for both tasks.

### 2.3 Predictors and preprocessing

Training-only observation filtering retained 106 continuous predictors for PH-Star and 105 for PH-VBP. PH-Star used healthcare-service (*n* = 84), insurance/cost (*n* = 9), and safety/medication-communication proxy (*n* = 13) views; PH-VBP retained 83, 9, and 13 variables, respectively. The last view contained six transformed healthcare-associated-infection standardized infection ratios, six corresponding CMS “Compared to National” comparison indicators, and the HCAHPS communication-about-medicines linear score. The 12 infection-related variables came from the same CMS healthcare-associated-infection table but represented distinct numeric and categorical-comparison fields. This view is a reporting proxy and not a direct inventory of pharmacy operations. Full variable names and engineered source families are given in Supplementary Table S1.

All preprocessing parameters were learned from training observations. Numeric source strings were parsed using CMS missing-value codes; measures with fewer than five observed training values were removed. Favorable direction was harmonized before scaling: Adverse events, readmissions, long waits, and payments were multiplied by −1; infection ratios used −log[max(SIR, 10^−4^)]; higher favorable scores retained their sign. Each continuous feature was winsorized at its training 1st and 99th percentiles, median-imputed using the training median, and standardized with the training mean and standard deviation. A binary missing indicator was retained before imputation. The observed test missing fractions were 0.261, 0.195, and 0.306 for the healthcare, insurance, and proxy views, respectively. In snapshots after August 2025, the CMS payment-and-value file used in development was absent; only Medicare spending per beneficiary remained observed in the nine-variable insurance/cost view. The post-development schema audit therefore recorded 1/9 insurance/cost variables with any observation and an 88.9% cell-missing rate. Missing variables were processed through training medians fixed during development and explicit missingness indicators; they were not reconstructed from later outcomes.

Stable context comprised one-hot encodings of state, county, hospital type, ownership, emergency-service status, electronic-health-record criteria, birthing-friendly designation, and four service availability flags (309 columns after training encoding). Unknown validation or test categories mapped to all-zero columns. Release time was encoded deterministically in 16 dimensions using eight sine/cosine pairs, as defined in Equation 2:


$$\begin{array}{ccc} e_{2j}(t) & = & \sin\text{​}\left(t/10000^{2j/16}\right),\\ e_{2j+1}(t) & = & \cos\text{​}\left(t/10000^{2j/16}\right),\;j=0,\ldots ,7, \end{array}$$ (2)

where *t* was the integer release position from the first downloaded snapshot. This encoding had no learned lookup table and therefore produced a defined vector for later releases.

### 2.4 Cross-sectional relations and temporal pairs

The PH-Star model was specified before evaluation with exactly three undirected cross-sectional graph relations at each feature release. Symmetric normalization included diagonal self-normalization, and *k* = 5 was used where neighbor selection was required. PH-VBP architecture search compared this predefined graph, graph matrices without diagonal self-loops, adaptive relation weighting at *k* = 3, 5, 8, and a graph bypass. The graph bypass was selected on FY2025. Under this configuration, all three adjacency matrices were represented as zero during training, early stopping, and inference. The variables and construction rules for the cross-sectional relations and temporal positive pairs are summarized in Table 2.

**Table 2:** Relation variables and edge rules.

Relation	Variables	Edge rule
Geographic	State, county, ZIP-derived latitude/longitude	Connect all hospitals in the same non-missing county. If a hospital has no same-county peer, connect its five nearest same-state hospitals by great-circle distance.
Organizational peer	Hospital type, ownership, emergency services, cardiac, surgical, emergency, and patient-survey service flags	Restrict candidates to matching type and ownership. Rank by Hamming distance over four service flags plus a 0.25 emergency-service mismatch penalty; connect five nearest candidates.
Performance similarity	Training-retained standardized continuous predictors	At release *t*, compute cosine distance using predictors from *t*−1 and connect five nearest hospitals. A hospital absent at *t*−1 uses its current predictors as a prespecified fallback.
Temporal positive	Facility identifier and consecutive task-specific period	Pair the same hospital in consecutive task-specific fitting periods: release-level for PH-Star and fiscal-year-level for the 2-year PH-VBP refit. Eligible negatives are different hospitals outside the anchor's type–ownership stratum.

Temporal pairs contribute to the loss and are not a fourth adjacency matrix. Performance similarity uses a prior release to avoid concurrent-target information.

ZIP-code centroids were obtained with pgeocode 0.5.0 from the US GeoNames postal-code database. The prior-release fallback affected 4 of 1,849 development-panel rows (0.22%). Training edge Jaccard indices were 0.0108 for geographic–peer, 0.0356 for geographic–performance, and 0.0147 for peer–performance, showing little overlap. PH-Star training contained 620 consecutive positive temporal pairs. The FY2024-only PH-VBP selection fit contained no within-training fiscal-year pair, so its temporal-contrastive term was zero. The 2-year historical refit contained 284 hospitals observed in both FY2024 and FY2025, permitting 284 consecutive annual positive pairs while retaining the selected graph bypass.

For an anchor *i*, positive *i*^+^, normalized projection *z*, eligible-negative set *N*_*i*_, and temperature γ = 0.1, temporal contrastive loss was defined in Equation 3 as


$${ℒ}_{\text{temp}}=-\frac{1}{\left|A\right|}\sum_{i\in A}\log\frac{\exp(\frac{z_{i}^{⊤}z_{i+}}{\gamma })}{\exp(\frac{z_{i}^{⊤}z_{i+}}{\gamma })+\sum_{j\in N_{i}}\exp(\frac{z_{i}^{⊤}z_{j}}{\gamma })}.$$ (3)

The denominator contains one positive and all eligible negatives. Validation and test pairs were not used for fitting.

### 2.5 HIOP-GL architecture and objective

For view *v*, standardized values *x*_*v*_, missingness mask *m*_*v*_, and context *c*, the feature-wise gate and encoder were defined in Equations 4–6 as


$$\begin{array}{lll} g_{v} & = & \sigma \left(a_{v}+b_{v}⊙m_{v}\right), \end{array}$$ (4)

$$\begin{array}{lll} h_{v}^{\left(1\right)} & = & Dropout\left\{\text{ReLU}\left[\text{LN}\left(W_{v}^{\left(1\right)}\left[g_{v}⊙x_{v};m_{v};c\right]+d_{v}^{\left(1\right)}\right)\right]\right\}, \end{array}$$ (5)

$$\begin{array}{lll} h_{v} & = & Dropout\left\{\text{ReLU}\left[\text{LN}\left(W_{v}^{\left(2\right)}h_{v}^{\left(1\right)}+d_{v}^{\left(2\right)}\right)\right]\right\}. \end{array}$$ (6)

Gate parameters were initialized at *a*_*v*_ = 2 and *b*_*v*_ = −2. Both layers had width 128 and dropout 0.15. View-specific scalar attention fused the three encodings. For PH-Star, global softmax relation weights combined self, geographic, peer, and prior-performance messages. The PH-VBP graph-bypass path retained the same encoder but set relation messages to zero.

The ordinal head produced a latent score *s*_*i*_ and four ordered thresholds. With θ_1_ unconstrained, the remaining thresholds were defined in Equation 7 as


$$\begin{array}{l} \theta_{k}=\theta_{1}+\overset{k}{\underset{r=2}{\sum }}\left\{\mathrm{softplus}\left(u_{r}\right)+10^{-4}\right\},\;k=2,3,4. \end{array}$$ (7)

Exceedance probabilities were *P*(*y*_*i*_>*k*) = σ(*s*_*i*_−θ_*k*_), and adjacent differences gave five class probabilities. PH-VBP additionally used a linear continuous head trained against standardized TPS with smooth-L1 loss. Its primary prediction was the continuous TPS transformed back to the original scale and mapped through the cutoffs defined in Equation 1.

Twenty-seven favorable-direction predictors received soft monotonic regularization on the ordinal latent score. The complete feature list, transformation, expected sign, and rationale appear in Supplementary Table S2. The PH-Star objective was defined in Equation 8 as


$$\begin{array}{l} L_{\text{Star}}=L_{\text{ord}}+0.10L_{\text{temp}}+0.05L_{\text{mono}}+10^{-4}||\Theta |{|}_{2}^{2}. \end{array}$$ (8)

The selected PH-VBP objective was defined in Equation 9 as


$${ℒ}_{\text{VBP}}={ℒ}_{\text{ord}}+0.50{ℒ}_{\text{TPS}}+0.10{ℒ}_{\text{tier}}+0.10{ℒ}_{\text{temp}}+0.05{ℒ}_{\text{mono}}+10^{-4}\|\Theta {\|}_{2}^{2},$$ (9)

where ℒ_tier_ was mean squared error between normalized expected and observed tiers. The temporal term was zero in the FY2024-only selection fit and active for 284 same-hospital annual pairs in the 2-year historical refit. PH-VBP early stopping used QWK after continuous TPS predictions were mapped through the cutoffs fixed from FY2024. The monotonic term constrains the parallel ordinal score, not the continuous TPS output, and therefore is not a monotonicity guarantee for PH-VBP.

The nominal-head ablation used ordinary cross-entropy, which does not explicitly encode the distance between ordered tiers. Its class objective therefore treats tier identities without an ordinal misclassification cost.

### 2.6 Comparators, tuning, and computation

PH-Star comparators were ordinal logistic regression, random forest, XGBoost, LightGBM, CatBoost, TabPFN v1, and ExcelFormer. Supplementary Methods S1 and Table M1 report the evaluated search grids, selected hyperparameters, and fixed settings, including the PyTorch Frame ExcelFormer implementation. Candidate models were ranked by validation QWK, with class-averaged Brier score as tie-breaker; within-fit stopping rules are specified separately in Supplementary Methods S1. The TabPFN v1 comparator used the first-generation classifier from tabpfn 0.1.11 (15) and the top 100 training-selected features.

Annual PH-VBP comparisons used continuous-TPS HistGradientBoosting, random forest, ExtraTrees, XGBoost, LightGBM, and CatBoost regressors, each mapped through the same cutoffs fixed from FY2024. These regressors used the fixed configurations listed in Supplementary Table M2, with no within-regressor hyperparameter search. HIOP candidates varied graph use, relation weighting, *k*, continuous-loss weight, monotonic regularization, hidden width, and dropout. Configuration selection used FY2025 only. The selected path used no graph messages, continuous-loss weight 0.50, width 128, dropout 0.15, AdamW at 10^−3^, at most 150 epochs, and patience 25. The best FY2025 epochs for seeds 20240701–20240705 were 11, 16, 11, 9, and 12. These counts were held constant when each corresponding seed was refitted on pooled FY2024–FY2025 records; no FY2026-based early stopping was performed.

The five seeds were 20240701–20240705. The PH-Star model had 282,207 trainable parameters; its previously reported mean training time was 70.2 ± 3.5 s on a four-core/eight-thread Windows 10 CPU with 16 GB RAM. In a fresh five-seed profile on the same environment, the annual PH-VBP path had 280,418 parameters, trained in 3.53 ± 0.46 s, and stopped at epoch 11.8 ± 2.6. The computing environment and software settings appear in Supplementary Methods S1, Table S4d reports parameter count, training time, inference time, peak memory, and model size. Additional methods and results are provided in Supplementary Data Sheet 1, with model checkpoints in Supplementary Data Sheets 2 and 3 and the main data and code package in Supplementary Data Sheet 4.

### 2.7 Evaluation and statistical analysis

Primary metrics were Macro-F1, QWK (30), tier-distance mean absolute error $\text{MA}{\text{E}}_{\text{T}}=n^{-1}\underset{i}{\sum }|y_{i}-{ŷ}_{i}|$, and low-tier recall, defined as the arithmetic mean of the separate recalls for tiers 1 and 2. Class-averaged Brier score, 10-bin Expected Calibration Error (ECE), and Negative Log-Likelihood (NLL) were retained before and after post-hoc temperature scaling for probabilistic PH-Star analyses (31). The thresholded continuous PH-VBP output is deterministic and was not presented as a calibrated probability model.

Mean ± SD summarizes five independently fitted models. Five-seed ensemble PH-Star probabilities were averaged before classification; PH-VBP TPS predictions were averaged before applying the cutoffs defined in FY2024. Hospital-block bootstrap resampled unique hospitals with replacement and retained all releases. The original benchmark used 1,000 resamples; post-development paired comparisons used 2,000. Percentile intervals and paired differences used the same hospital draw for both methods. PH-VBP ablation intervals used 2,000 paired hospital resamples on the 301-hospital FY2025 tuning cohort. They compare five-seed ensembles, condition on model selection, and are reported as descriptive intervals without multiplicity adjustment (Supplementary Table S11c). Two-sided empirical *p*-values used 2min{*P*(Δ ≤ 0), *P*(Δ≥0)} and Holm adjustment within task and metric (32).

PH-Star retained the prespecified temporal test, hospital-grouped validation, masking and label-perturbation tests, complete-view outage comparisons, calibration, release and subgroup analyses, monotonic perturbations, and Integrated Gradients. Post-development temporal validation applied the training transformations and selected models, all held constant after development, to four later release pairs. For PH-VBP, FY2024–FY2025 model choices were fixed in advance of the pooled historical refit and FY2026 evaluation. A five-fold hospital-disjoint temporal design assigned facility identifiers by a deterministic facility-ID mapping modulo five. For each fold, all FY2024–FY2025 rows from its FY2026 evaluation hospitals were excluded before preprocessing and fitting. A second design left out each of four Census regions in turn. Historical hospitals in the held-out region were excluded, and the model predicted FY2026 hospitals in that region. Fold and region assignment used identifiers and states, not outcomes. Concatenated out-of-group predictions covered all 308 FY2026 hospitals exactly once. These are institution-separated and geographic-region-separated analyses within the same CMS reporting system, not validation by an independent data owner.

With every PH-Star model choice and weight held constant, three supplementary external checks were conducted without model or threshold selection. They answer different questions and were interpreted separately. The first tests ownership transport for the same CMS outcome, not data-owner independence. The five-seed ensemble was applied without refitting to the 6 October 2023 CMS feature release for 5,446 US hospitals. Graph relations were reconstructed from predictor and facility information within that scoring release without using external outcomes. The ownership-external cohort comprised California non-public acute-care hospitals, which had zero facility-ID overlap with the public-hospital development, validation, and temporal-test panel. Linking 6 October 2023 features to 31 January 2024 PH-Star retained 229 hospitals with reported ratings. Percentile intervals used 10,000 hospital bootstrap resamples.

The second check tests criterion concordance with HCAI, not reproduction of PH-Star. CMS Certification Numbers were linked to HCAI identifiers only through the official crosswalk. A 2023 multi-measure criterion averaged all reportable HCAI ratings after coding Better as 1, As Expected as 0, and Worse as −1; at least five ratings were required. A mortality-specific sensitivity used 2024 HCAI risk-adjusted mortality rates for acute myocardial infarction, acute stroke, gastrointestinal hemorrhage, heart failure, hip fracture, and pneumonia; reversed within-condition percentiles were averaged when at least four conditions were available.

The third check changes both state authority and criterion. We linked non-public New York hospitals scored by the unchanged model using 6 October 2023 features to 2024 NYSDOH IQI composites by county and normalized hospital name. The primary analysis required an exact normalized-name match, county agreement, and a unique one-to-one link in both sources; ambiguous campus or health-system links were rejected. Both IQI90 (mortality for selected procedures) and IQI91 (mortality for selected conditions) were required. Their composite quality criterion was the negative mean log of the two reported risk-adjusted composite measures, so larger values indicated lower mortality. A sensitivity admitted unique high-confidence name matches with similarity at least 0.86 and a margin of at least 0.08 over the next candidate. We assessed linkage selection by comparing the model-score distribution and hospital-type and ownership composition of the 57 exact-linked hospitals with the other 85 eligible non-public New York hospitals. Spearman's correlation compared each state criterion with the expected PH-Star tier from the held-constant model. Intervals used 10,000 hospital bootstrap resamples; two-sided permutation tests used 99,999 shuffles. The California multi-measure criterion, California mortality sensitivity, and primary New York composite were Holm-adjusted together. A more conservative multiplicity sensitivity additionally Holm-adjusted all six model-to-external-criterion associations: the two California analyses, New York exact and expanded links, and both New York components. The observed next-release PH-Star reference was descriptive and excluded from this family. These checks assess independent-authority criteria; they do not constitute independent-data-owner same-outcome validation or prospective deployment studies.

### 2.8 Interpretability and monotonicity diagnostics

For PH-Star, Integrated Gradients with 32 integration steps used the training median as baseline and explained the latent ordinal score *s*_*i*_ (33). Absolute attributions were aggregated first by feature and then by view. Monotonicity was tested by increasing each of the 27 constrained standardized predictors by 0.1 training SD while holding the graph fixed. We report mean latent-score change per 1 SD and the fraction of hospital-feature perturbations producing a negative score change. Hospital-block bootstrap intervals quantified uncertainty. These are associational model diagnostics, not causal effects.

## 3 Results

### 3.1 PH-Star temporal benchmark

In the final temporal-validation analysis, the PH-Star model fixed before temporal testing yielded QWK 0.816 ± 0.009, Macro-F1 0.584 ± 0.036, MAE_T_ 0.455 ± 0.037, and low-tier recall 0.600 ± 0.015 across five fits (Table 3). The final five-seed ensemble QWK was 0.824. In the earlier benchmark analysis, whose per-tier results and confusion matrices are presented in Supplementary Tables S9a and S10a, the HIOP-GL ensemble QWK also rounded to 0.824; its differences from random forest (0.816) and TabPFN v1 (0.801) were not significant after Holm adjustment. Random forest retained higher exact-class Macro-F1 and lower tier-distance error, whereas TabPFN v1 retained higher low-tier recall.

**Table 3:** PH-Star temporal-test benchmark.

Method	Macro-F1	QWK	MAE_T_	Brier	Low-tier recall
Ordinal logistic	0.569 ± 0.000	0.780 ± 0.000	0.493 ± 0.000	0.1224 ± 0.0000	0.572 ± 0.000
Random forest	**0.682 ± 0.014**	0.805 ± 0.016	**0.324 ± 0.019**	0.1085 ± 0.0005	0.488 ± 0.018
CatBoost	0.619 ± 0.008	0.764 ± 0.016	0.395 ± 0.014	**0.0958 ± 0.0010**	0.421 ± 0.011
TabPFN v1	0.589 ± 0.008	0.795 ± 0.003	0.467 ± 0.007	0.1113 ± 0.0020	**0.639 ± 0.023**
ExcelFormer	0.512 ± 0.053	0.703 ± 0.028	0.557 ± 0.032	0.1218 ± 0.0061	0.455 ± 0.076
HIOP-GL	0.584 ± 0.036	**0.816 ± 0.009**	0.455 ± 0.037	0.1173 ± 0.0027	0.600 ± 0.015

Every numerical entry is the mean ± SD across five fits. Brier is class-averaged and calculated before temperature scaling. Bold marks the favorable mean, not statistical superiority.

### 3.2 Annual PH-VBP tuning and targeted ablation

On FY2025 tuning, the selected continuous-TPS graph-bypass path achieved QWK 0.716 ± 0.023, Macro-F1 0.413 ± 0.008, MAE_T_ 0.743 ± 0.035, and low-tier recall 0.456 ± 0.055. The FY2025 architecture and view ablations are summarized in Table 4. The five-seed TPS ensemble reached QWK 0.759 (95% CI 0.713–0.804). The strongest continuous baseline, HistGradientBoosting, reached QWK 0.697, Macro-F1 0.443, and MAE_T_ 0.704. The improvement was therefore specific to ordinal agreement; it did not extend to Macro-F1 or tier-distance error in individual fits.

**Table 4:** FY2025 PH-VBP architecture and view ablations.

**Variant**	Macro-F1	QWK	MAE_T_	Low-tier recall
Selected graph bypass + TPS head	0.413 ± 0.008	0.716 ± 0.023	0.743 ± 0.035	0.456 ± 0.055
No monotonic regularization	0.441 ± 0.025	0.715 ± 0.026	0.728 ± 0.047	0.518 ± 0.053
Adaptive graph, k=8	0.419 ± 0.010	0.710 ± 0.018	0.753 ± 0.023	0.497 ± 0.017
No healthcare view	0.424 ± 0.010	0.702 ± 0.010	0.756 ± 0.017	0.444 ± 0.034
No insurance/cost view	0.411 ± 0.008	0.679 ± 0.018	0.779 ± 0.016	0.446 ± 0.057
Predefined graph, k=5	0.407 ± 0.022	0.678 ± 0.041	0.766 ± 0.021	0.441 ± 0.080
Ordinal head only	0.288 ± 0.034	0.642 ± 0.028	0.966 ± 0.073	0.460 ± 0.016
No safety/proxy view	0.340 ± 0.014	0.581 ± 0.030	0.902 ± 0.015	0.355 ± 0.045

Values are mean ± SD across five fits. All continuous-head variants apply cutoffs fixed from FY2024.

Across individual fits, selected PH-VBP QWK differed by less than 0.001 from the no-monotonic variant, which had higher Macro-F1 and low-tier recall. For the five-seed ensembles, paired QWK differences favored the selected path over the ordinal-only head by 0.124 (95% CI 0.077–0.171), removal of the safety/proxy view by 0.148 (0.096–0.205), and removal of the insurance/cost view by 0.060 (0.024–0.101). Differences from the adaptive graph and no-monotonic variants were 0.001 (−0.014 to 0.016) and 0.010 (−0.013 to 0.034), respectively. Supplementary Table S11c reports all four metrics for every paired comparison. These descriptive intervals characterize component sensitivity on the FY2025 tuning cohort; subsequent temporal performance is evaluated separately below.

### 3.3 Post-development temporal and separated-group validation

Across four later PH-Star release pairs, QWK was 0.757 ± 0.013 across fits and 0.768 (95% CI 0.733–0.798) for the ensemble (Figure 3). CatBoost and random forest reached 0.705 ± 0.009 and 0.680 ± 0.005. The paired ensemble QWK differences were 0.057 (95% CI 0.024–0.091; Holm *p* = 0.002) vs. CatBoost and 0.072 (0.038–0.108; *p* = 0.002) vs. random forest. Macro-F1 differences crossed zero. For these same 1,236 observations from 338 hospitals, the five-seed ensembles correctly classified 640 observations for HIOP-GL, 657 for CatBoost, and 646 for random forest. Supplementary Tables S9b and S10b give the complete per-tier results and confusion matrices for the post-development cohort. Supplementary Tables S9a and S10a report the earlier benchmark analysis of the original 603-observation temporal-test cohort. Exact-tier counts and QWK measure different aspects of performance because QWK also accounts for tier distance and the observed and predicted marginal distributions. For tier 1, HIOP-GL correctly classified 86 of 140 observations, compared with 14 for CatBoost and 17 for random forest; the corresponding recalls were 0.614, 0.100, and 0.121.

**Figure 3:**
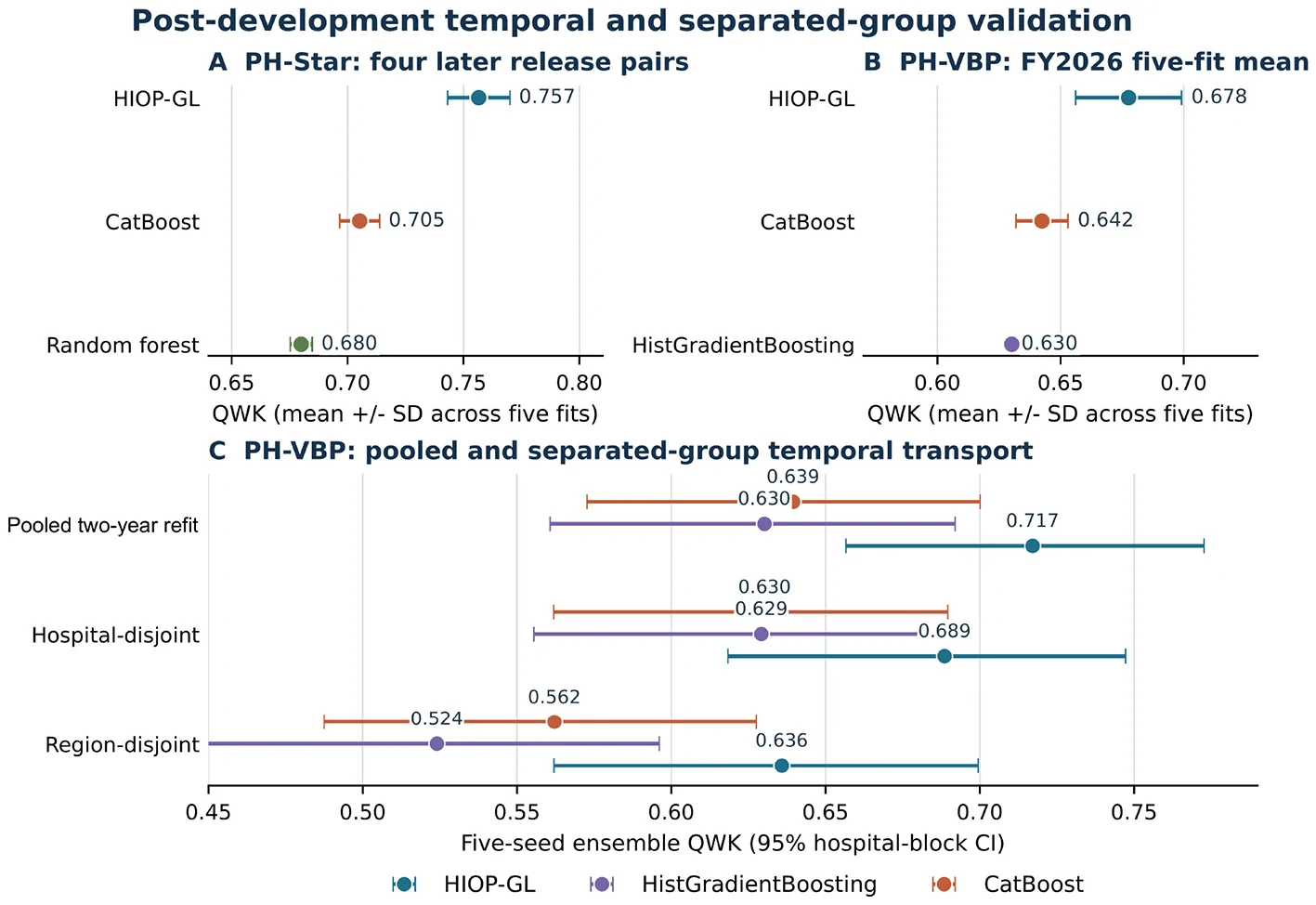
Post-development temporal and separated-group validation. (**A** and **B**) show mean QWK with SD across five fits for PH-Star over four later release pairs (1,236 observations, 338 hospitals) and 2-year-refit PH-VBP in FY2026 (308 hospitals). **(C)** Shows five-seed PH-VBP ensemble QWK with 95% hospital-block intervals for pooled historical refit, hospital-disjoint temporal cross-fitting, and Census-region-disjoint temporal cross-fitting. All evaluations use later snapshots from the same CMS reporting system.

The graph-bypass architecture, cutoffs, and seed-specific epoch counts were held constant after FY2025 tuning. Refitting on all 622 historical hospital-year records produced FY2026 PH-VBP QWK 0.678 ± 0.021 across five fits. HistGradientBoosting and CatBoost reached five-fit mean QWK 0.630 ± 0.000 and 0.642 ± 0.011, respectively (Figure 3B). The corresponding five-seed ensemble QWK values were 0.717 (95% CI 0.657–0.773) for HIOP-GL, 0.630 for HistGradientBoosting, and 0.639 for CatBoost. Paired ensemble QWK differences were 0.087 (0.043–0.134; Holm *p* = 0.002) vs. HistGradientBoosting and 0.077 (0.033–0.123; *p* = 0.002) vs. CatBoost. HIOP-GL ensemble MAE_T_ was 0.584, vs. 0.714 and 0.711, respectively. Macro-F1 was 0.402 for HIOP-GL and 0.409 for HistGradientBoosting; the paired interval crossed zero. Low-tier recall was 0.469 for HIOP-GL, 0.568 for HistGradientBoosting, and 0.469 for CatBoost, with both paired intervals crossing zero.

The hospital-disjoint temporal ensemble predicted each FY2026 hospital only with models fitted without that facility's FY2024–FY2025 records. It reached QWK 0.689 (95% CI 0.618–0.747). HistGradientBoosting and CatBoost reached 0.629 and 0.630; HIOP-GL-minus-comparator QWK intervals were −0.002 to 0.121 and 0.003 to 0.116, with Holm *p* = 0.072 for both. Leave-Census-region-out temporal prediction reached HIOP-GL QWK 0.636 (0.562–0.699), vs. 0.524 and 0.562. Paired QWK differences were 0.111 (0.041–0.178; Holm *p* = 0.002) and 0.072 (0.008–0.132; *p* = 0.032). These separated-group estimates reduce direct hospital and regional overlap but remain internal to the same CMS reporting system.

Both the target distribution and feature schema changed during transport. Mean TPS increased from 22.51 in FY2025 to 29.84 in FY2026; 48.1% of FY2026 hospitals consequently occupied tier 5 under the cutoffs fixed from FY2024. From August 2025 onward, only 1 of 9 development insurance/cost variables remained observed. Performance below the tuning period must therefore be interpreted in the context of both target and schema drift.

### 3.4 Post-development ownership transport and independent-authority criterion checks

Among 229 California non-public hospitals with a reported next-release PH-Star, the five-seed ensemble, held unchanged after development, achieved QWK 0.637 (95% CI 0.546–0.718), Macro-F1 0.441 (0.377–0.500), tier-distance MAE 0.721 (0.616–0.825), and low-tier recall 0.387 (0.259–0.517). None of these facilities appeared in the public-hospital development, validation, or temporal-test panel.

Official crosswalk linkage yielded 243 hospitals for the 2023 HCAI multi-measure performance criterion. The expected PH-Star tier from the held-constant model showed positive but modest concordance (ρ = 0.247, 95% CI 0.125–0.366; permutation *p* = 0.0002; three-criterion Holm *p* = 0.0006). The association remained positive when minimum criterion coverage varied from 3 to 12 rated measures (ρ = 0.241–0.334). In the 2024 HCAI mortality-specific sensitivity (*n* = 233), concordance was weaker and not significant (ρ = 0.096, 95% CI −0.035 to 0.229; permutation and Holm *p* = 0.144).

The NYSDOH source contained 185 hospitals with both 2024 mortality composites; 142 non-public New York hospitals had CMS-based scores from the unchanged model. After rejecting ambiguous or non-unique links, the primary exact-name-and-county analysis contained 57 hospitals. Their mean model-derived expected tier was 2.554 vs. 2.479 in the other 85 eligible hospitals (standardized mean difference 0.066; Mann–Whitney *p* = 0.464; Kolmogorov–Smirnov *p* = 0.545). These comparisons provided no evidence that exact linkage preferentially retained high-scoring hospitals. Hospital-type composition nevertheless differed: Exact links contained 78.9% acute-care and 21.1% critical-access hospitals, whereas unmatched hospitals contained 85.9% acute-care, 5.9% critical-access, 7.1% psychiatric, and 1.2% children's hospitals. The expected PH-Star tier from the held-constant model correlated with lower NYSDOH risk-adjusted mortality (ρ = 0.451, 95% CI 0.195–0.663; permutation *p* = 0.00048; three-criterion Holm *p* = 0.00096). Expanding to 67 unique high-confidence links gave ρ = 0.415 (0.161–0.628; permutation *p* = 0.0006). Component results were heterogeneous: Concordance was supported for mortality in selected conditions (IQI91; ρ = 0.441, 0.192–0.647) but not selected procedures (IQI90; ρ = 0.179, −0.138 to 0.464). In the six-association multiplicity sensitivity, Holm-adjusted *p*-values were 0.0012 for California broad performance, 0.0024 for the New York exact-link, expanded-link, and IQI91 associations, and 0.287 for both mortality associations that were unsupported. Across the state checks, concordance was supported for broad performance in California and joint mortality in New York without relying on selective significance reporting. However, the composition differences, unsupported California mortality analysis, and unsupported New York procedure component preclude a general mortality-validity claim.

These analyses address different transport questions. The California non-public cohort changes ownership and facility identity while retaining the CMS feature and outcome system; it estimates same-outcome ownership transport only. HCAI and NYSDOH change the reporting authority and criterion, and NYSDOH additionally changes the state and all-payer data process. Together, these sources assess multi-state cross-system construct concordance, not an independently collected PH-Star equivalent. Because all checks were added after model choices had been fixed and address non-equivalent questions, they remain supplementary boundary tests rather than confirmatory independent same-outcome validation.

### 3.5 PH-Star grouped, robustness, calibration, and interpretation analyses

The previously reported hospital-grouped PH-Star evaluation remained materially lower than the ordinary temporal test. Pooled QWK was 0.661 for HIOP-GL and 0.730 for ordinal logistic when every evaluated hospital was absent from its fold's development data. Among the 16 temporal-test hospitals absent from training, HIOP-GL QWK was 0.556 ± 0.048. These results do not support choosing the graph model solely for unseen-institution prediction. Ordinal logistic remains the stronger benchmark in the fully hospital-grouped PH-Star setting.

PH-Star robustness evidence was metric-dependent. Under 20 and 40% additional masking, HIOP-GL QWK was 0.793 ± 0.010 and 0.747 ± 0.008. When the healthcare view was entirely unavailable, retraining without that view exceeded exposing the full model to outage by QWK 0.282 (95% CI 0.214–0.354). With 10% adjacent-tier label perturbation, HIOP-GL QWK was 0.735 ± 0.085, below TabPFN v1 and CatBoost. Full low-tier-recall results, repetitions, corresponding-model outage tests, confusion matrices, and per-tier errors remain in the Supplementary material.

In a separate calibration analysis of the earlier PH-Star benchmark fits (603 temporal-test observations; five fits), temperature scaling changed HIOP-GL Brier from 0.1166 to 0.1151, ECE from 0.226 to 0.195, and NLL from 1.075 to 1.062; TabPFN v1 had lower pre-scaling ECE (0.036). These HIOP-GL calibration results use the earlier fit set underlying Supplementary Tables S9a and S10a. The final temporal-validation fits summarized in Table 3 and Supplementary Table S5 constitute a separate fit set. Supplementary Figure S1 shows the primary-seed reliability curves, and Supplementary Table S14 gives the corresponding five-fit calibration summaries. Region-specific PH-Star QWK ranged from 0.781 to 0.832. Monotonic perturbations produced a mean latent-score effect of 0.083 per 1 SD (95% CI 0.078–0.089), but 7.1% (6.3–7.9%) of local perturbations had the opposite sign. Integrated Gradients assigned 77.9%, 8.8%, and 13.3% of absolute attribution to healthcare, insurance, and proxy views. These diagnostics explain fitted scores and do not estimate causal effects.

### 3.6 Metric-conditional model comparison

HIOP-GL's advantage was most consistent for ordinal agreement in later-release evaluation. The original PH-Star temporal test also favored HIOP-GL in mean QWK. Random forest had higher Macro-F1 (0.682 vs. 0.584) and lower tier-distance MAE (0.324 vs. 0.455), while TabPFN v1 had higher low-tier recall (0.639 vs. 0.600) and lower pre-scaling ECE. The ensemble QWK differences from both comparators were not significant after multiplicity adjustment. In the fully hospital-grouped PH-Star evaluation, ordinal logistic exceeded HIOP-GL by 0.069 QWK. In FY2026 PH-VBP, HIOP-GL improved ensemble QWK and tier-distance MAE relative to HistGradientBoosting. However, Macro-F1 was 0.402 vs. 0.409 and low-tier recall was 0.469 vs. 0.568, without supported paired differences. These comparisons locate the benefit of the outcome-specific architecture in ordinal agreement under temporal transport. Exact-tier classification, low-tier detection, and transfer to entirely new institutions require their own performance criteria.

## 4 Discussion

The central finding is improved ordinal agreement during temporal transport with an architecture adapted to each outcome. Across four later PH-Star release pairs, the HIOP-GL ensemble exceeded CatBoost and random forest in QWK, with paired intervals above zero after Holm adjustment. The annual PH-VBP path also improved FY2026 QWK relative to the selected continuous baselines in pooled and region-disjoint evaluation. The hospital-disjoint estimate remained favorable, although its adjusted comparison was inconclusive. These results connect the modeling choice to the prediction target: PH-Star retains an ordinal graph path, whereas annual PH-VBP uses a continuous-TPS graph bypass.

Matching the analysis unit to outcome frequency is central to this interpretation. HVBP TPS is updated annually, so one release pair per fiscal year represents an outcome update without counting repeated quarterly values as independent information. Pooling FY2024 and FY2025 after architecture and epoch counts were fixed increased the historical fitting sample while preserving FY2026 for evaluation. The selected graph bypass identifies the useful PH-VBP representation as a multi-view encoder with a continuous head; annual temporal pairs enter the historical refit's training loss. This distinction gives the two tasks a common representation while allowing their prediction paths to differ.

Later-release performance was metric and cohort-dependent. HIOP ensemble QWK exceeded the selected conventional baselines for both outcomes, while several Macro-F1 and low-tier-recall differences were not supported. Ordinal logistic also remained stronger in the fully hospital-grouped PH-Star analysis. Model choice should therefore follow the decision metric and target cohort rather than architecture alone. The supported advantage concerns QWK-focused retrospective stratification; the complete metric and cohort comparisons identify its practical scope. PH-VBP hospital-disjoint and region-disjoint cross-fitting removed direct overlap along the designated grouping dimension. These designs did not introduce an independent health system; the reporting authority and data-generating process remained unchanged. Distinguishing temporal evaluation from deployment to new institutions or reporting systems is essential because healthcare models can fail under dataset shift even when development performance is strong (34, 35). The California non-public cohort adds a zero-overlap ownership setting for the same CMS outcome, with lower agreement than the public-hospital temporal test. HCAI and NYSDOH add two independent state authorities and different data processes, but they evaluate construct concordance rather than the same outcome. The supported California broad-performance and New York joint-mortality results provide evidence for multi-state boundary assessment and survived a conservative six-association Holm sensitivity. The unsupported California mortality sensitivity and NYSDOH procedure component show that concordance is criterion-dependent. Similar model-score distributions in matched and unmatched eligible New York hospitals reduce concern that linkage selected on the model score, but differences in hospital-type composition remain a transportability limitation. These checks do not fill the independent same-outcome evidence gap. PH-VBP tier prevalence also shifted sharply because FY2024 cutoffs were deliberately kept fixed, and the insurance/cost view lost eight of nine variables. The FY2026 evaluation therefore assessed preprocessing held constant from development under an observed schema change; its estimate should not be read as performance under stable measurement.

Other PH-Star analyses identify additional limits. Grouped and new-hospital performance was lower, additional missingness reduced agreement, temperature scaling provided limited improvement, and soft monotonic regularization did not eliminate local violations. Integrated Gradients and view ablations identify model dependence, not effects of changing hospital practice.

### 4.1 Limitations

Primary predictors and target outcomes originated from the CMS administrative reporting system. The four later PH-Star pairs and FY2026 PH-VBP pair are temporally later than development. Hospital-disjoint and Census-region-disjoint PH-VBP cross-fitting remove overlap by the specified grouping dimension, but they are not independent by data owner or reporting process. The California non-public cohort remains a same-CMS-outcome analysis. HCAI and NYSDOH use independently maintained but different criteria rather than an independently collected PH-Star equivalent. The HCAI ratings are retrospective and heterogeneous in measure availability; its mortality sensitivity was not significant. The NYSDOH primary link was intentionally restricted to 57 exact-name-and-county matches, and concordance differed between its procedure and condition components. Matched and unmatched eligible hospitals had similar model-score distributions. However, the exact-link cohort overrepresented critical-access hospitals and contained no psychiatric or children's hospitals, so the NYSDOH result is not representative of every eligible hospital type. These analyses do not replace prospective multisystem validation. Regional sample sizes were unequal, including 18 Northeast and 167 South FY2026 hospitals. The common-outcome cohort excludes hospitals without both reported outcomes. Program rules, measure definitions, file availability, suppression, coding, and denominator stability can change. PH-VBP spans only three fiscal-year outcomes. The refit can form annual temporal pairs for 284 repeated hospitals but cannot characterize longer trajectories. Thresholded TPS predictions do not provide calibrated class probabilities, and low-tier recall was not improved relative to HistGradientBoosting. Subgroup analyses do not constitute a fairness audit. The proxy view does not represent detailed pharmacy operations. Finally, this retrospective study did not evaluate workflow decisions, costs, patient outcomes, or causal impact.

## 5 Conclusion

HIOP-GL improved ordinal agreement in later-release hospital performance stratification by adapting the prediction path to the outcome. PH-Star used an ordinal graph path; annual PH-VBP used continuous-TPS prediction with a graph bypass and a 2-year historical refit. The most consistent gains were in temporal QWK, with metric-specific trade-offs reported for exact-tier classification and low-tier recall. Hospital- and Census-region-disjoint PH-VBP analyses extend the assessment across groups within CMS, while the zero-overlap non-public cohort tests same-outcome ownership transport. Concordance with HCAI and NYSDOH criteria adds evidence from independent reporting authorities, subject to their different outcome definitions. Prospective evaluation of the same outcomes across independent health systems is the next step toward operational use.

## Data Availability

The original contributions presented in the study are included in the article/Supplementary material, further inquiries can be directed to the corresponding author.

## Supplementary material

Data Sheet 1 Supplementary methods and results. Implementation and reproducibility details, comparator configurations, predictor specifications, cohort and data-integrity audits, validation and sensitivity analyses, and supplementary performance, calibration, and interpretability results, including Supplementary Figure S1.

Data Sheet 2 Model checkpoints and configuration metadata for four annual PH-VBP ablation variants: removal of the healthcare-service view, insurance/cost view, safety/medication-communication proxy view, and monotonic regularization. Five seeds per variant are provided, for a total of 20 checkpoints.

Data Sheet 3 Model checkpoints and configuration metadata for the selected graph-bypass continuous-TPS pathway, the adaptive-graph pathway with k = 8 and a continuous-TPS head, the predefined-graph pathway with k = 5 and a continuous-TPS head, and the ordinal-head-only pathway. Five seeds per configuration are provided, for a total of 20 checkpoints.

Data Sheet 4 Main supplementary data and code package. Processed hospital-level data, analysis code, an executed numerical-audit notebook, saved predictions, numerical result tables, and selected model checkpoints for the PH-Star and annual PH-VBP analyses. Complementary annual PH-VBP configuration and ablation checkpoints are supplied in Data Sheets 2 and 3.

Supplementary Figure S1 PH-Star reliability curves before and after validation-fitted temperature scaling. Curves show confidence-bin accuracy for HIOP-GL, random forest, and CatBoost; empty bins are omitted. The diagonal denotes perfect agreement between mean confidence and observed accuracy. PH-VBP is omitted because the selected pathway predicts continuous TPS followed by deterministic locked cutoffs rather than class probabilities. The curves show the primary seed (20240701) from the archived calibration analysis; Supplementary Table S14 summarizes all five seeds. The HIOP-GL curve uses the earlier benchmark fit set described in Supplementary Table S14 and is distinct from the final temporal-validation fits summarized in Table 3 and Supplementary Table S5.
