# Supplementary material for: Task-adaptive multi-view learning with ordinal graph and continuous-score paths for public hospital performance stratification
Source: Front Public Health. 2026 Oct 30;14:1890759. doi: 10.3389/fpubh.2026.1890759 (PMC13760696; doi:10.3389/fpubh.2026.1890759)
Supplement: Supplementary file 1 [file Data_Sheet_1.pdf]

Supplementary Methods S1: implementation and reproducibility

The analysis used Python 3.11.9 on Windows 10 with four physical CPU cores, eight logical threads, and 16 GB RAM. Principal packages were PyTorch 2.5.1 (CPU), scikit-learn 1.9.0, XGBoost 3.2.0, LightGBM 4.7.0, CatBoost 1.2.10, TabPFN v1 (package `tabpfn` 0.1.11), pandas 3.0.5, NumPy 2.4.6, SciPy 1.17.1, Captum 0.9.0, and `pgeocode` 0.5.0. HIOP-GL used hidden width 128, dropout 0.15, AdamW learning rate 10^{-3}, and weight decay 10^{-4}. PH-Star used batch size 256, at most 200 epochs, patience 30, temporal weight 0.10, and monotonic weight 0.05. PH-VBP used at most 150 epochs, patience 25, a continuous-TPS loss weight of 0.50, a tier loss weight of 0.10, a temporal weight of 0.10, and monotonic weight 0.05. The PH-VBP temporal term was zero in the FY2024-only selection fit and active for 284 annual pairs in the locked refit. Seeds were 20240701–20240705.

PH-Star model selection used the original validation split. PH-VBP architecture, loss, and epoch selection used FY2024 development and FY2025 tuning only. The seed-specific selected epochs were 11, 16, 11, 9, and 12. After locking these choices, final PH-VBP models were fitted for the corresponding fixed epoch counts on pooled FY2024–FY2025 records and evaluated in FY2026; no outcome-based early stopping used FY2026. Five-seed ensemble PH-Star probabilities were averaged before classification, whereas PH-VBP TPS predictions were averaged before applying the FY2024-locked cutoffs. Hospital-block intervals resampled facilities and retained all rows belonging to each sampled hospital. Post-development paired model comparisons used identical hospital draws, 2,000 resamples, two-sided empirical p values, and Holm adjustment within design and metric. The PH-VBP component intervals in Table S11c are descriptive, unadjusted percentile intervals on the FY2025 tuning cohort; mean ± SD across fits remains separately reported in Table S4b.

Hospital-disjoint PH-VBP temporal cross-fitting assigned facility identifiers by a deterministic facility-ID mapping modulo five and removed every historical row for each fold's FY2026 evaluation hospitals before preprocessing. Census-region-disjoint cross-fitting removed all historical hospitals in each held-out region before predicting FY2026 hospitals from that region. The two concatenated out-of-group prediction sets each covered all 308 FY2026 hospitals exactly once. Assignment used facility identifiers and states, not outcome values. The accompanying executed notebook reads the saved CSV files and reproduces the numerical summary tables without refitting the models.

Comparator search spaces and fitted settings

PH-Star selection. The five conventional classifiers used 106 standardized continuous values, 106 missingness indicators, 309 one-hot context columns, and 16 release-time components. Preprocessing was fitted on the 945 training observations. Each grid in Table M1 was evaluated on the original 301-observation validation split using seed 20240701. Configurations were ranked by quadratic weighted kappa (QWK) of the maximum-probability class, with lower class-averaged Brier score breaking ties. Brier score averaged squared probability error over observations and all five classes. Complete ties retained the first evaluated configuration. Selected hyperparameters were held fixed across fits with seeds 20240701–20240705.

Table M1. PH-Star comparator searches and selected settings on the original validation split. Grid entries denote Cartesian products.

Comparator	Evaluated search space	Selected setting	Fixed settings
Ordinal logistic	$\alpha \in \{0.01, 0.1, 1, 10\}$; 4 configurations	$\alpha = 1$	All-threshold logistic model, `mord.LogisticAT` (0.7); squared L_2 penalty; maximum 10,000 optimizer iterations.
Random forest	Maximum depth $\in$ {unlimited, 12}; minimum leaf size $\in \{1, 5\}$; 4 configurations	Unlimited depth; minimum leaf size 1	500 trees; square-root feature sampling.
XGBoost	Maximum depth $\in \{3, 6\}$; learning rate $\in \{0.03, 0.1\}$; 4 configurations	Depth 6; learning rate 0.1	Maximum 1,000 boosting rounds; row and column fractions 0.8; histogram tree method; five-class `multi:softprob` objective.
LightGBM	Number of leaves $\in \{15, 31\}$; learning rate $\in \{0.03, 0.1\}$; 4 configurations	31 leaves; learning rate 0.1	Maximum 1,000 boosting rounds; column fraction 0.8; multiclass objective; row-subsampling setting as noted below.
CatBoost	Depth $\in \{4, 6\}$; learning rate $\in \{0.03, 0.1\}$; 4 configurations	Depth 6; learning rate 0.03	Maximum 1,000 iterations; `MultiClass` loss and evaluation metric.
TabPFN v1	One fixed configuration	Fixed settings at right	`tabpfn` 0.1.11; CPU inference; 3 ensemble configurations; feature subsampling enabled; inference batch size 1; top 100 training-selected columns.

Comparator	Evaluated search space	Selected setting	Fixed settings
ExcelFormer	Channel width $\in \{32, 64\}$; layers $\in \{1, 2\}$; 4 configurations	Width 64; 1 layer	PyTorch Frame 0.3.0; 4 attention heads; 5 outputs; top 100 training-selected columns; training settings below.

XGBoost, LightGBM, and CatBoost used validation multiclass log loss for within-fit early stopping, with patience 30; QWK and Brier score ranked the resulting candidates. Both LightGBM pathways specified `subsample=0.8` and retained `subsample_freq=0`, leaving row bagging inactive. Other estimator arguments retained their installed-library defaults. Grid selection used pre-scaling probabilities. Subsequent calibration fitted a positive scalar temperature by validation negative log likelihood, with $\log T \in [-3, 3]$. In the hospital-grouped PH-Star analysis, the same conventional-classifier grids were evaluated separately within each fold, excluding held-out hospitals from both training and validation.

TabPFN v1 and ExcelFormer implementation. For both methods, the top 100 columns of the concatenated matrix were selected and ordered by decreasing training-only mutual information with PH-Star. Selection used `mutual_info_classif`, with `discrete_features=False`, random state 20240701, and stable ordering of ties. The feature set was held fixed across seeds. TabPFN v1 used the pretrained classifier in inference-only mode.

ExcelFormer used `torch_frame.nn.ExcelFormer` from PyTorch Frame 0.3.0 (`https://github.com/pyg-team/pytorch-frame`). All selected columns, including one-hot context, were supplied as numerical inputs with training-derived means and standard deviations. Directed inter-feature attention dropout was 0.3; intra-feature and residual dropout were zero. Feature and hidden mixup were disabled (`mixup=None`). Training used multiclass cross-entropy and Adam with learning rate 10^{-3}, zero weight decay, $\beta_1 = 0.9$, $\beta_2 = 0.999$, and $\epsilon = 10^{-8}$. Batch size was 256, the learning rate was multiplied by 0.95 after each epoch, and gradient norms were clipped at 5. Training ran on CPU for at most 50 epochs, with patience 10 under the QWK/Brier ordering above. The best validation checkpoint was restored. Selected epochs for seeds 20240701–20240705 were 33, 28, 35, 14, and 23, respectively.

Continuous PH-VBP comparators. The six regressors in Table M2 each used one fixed configuration. They were fitted to continuous TPS in FY2024 ($n = 321$), using training-fitted preprocessing and the full concatenated predictor matrix, and evaluated on FY2025 ($n = 301$) with seeds 20240701–20240705. Configurations were compared by mean QWK after mapping TPS predictions through the FY2024-locked cutoffs; TPS MAE and RMSE were also reported. All regressors used the iteration or tree counts in Table M2 without validation-based early stopping. HistGradientBoosting retained `early_stopping='auto'`, which was inactive at these sample sizes.

Table M2. Fixed configurations of the continuous-TPS PH-VBP regressors. Each row is one evaluated configuration.

Regressor	Fixed configuration
HistGradientBoosting	Learning rate 0.05; 300 iterations; maximum 15 leaf nodes; L_2 regularization 1.0; squared-error loss; default minimum leaf size 20.
Random forest	500 trees; feature fraction 0.5; minimum leaf size 4; unlimited depth; squared-error criterion; bootstrap sampling enabled.
ExtraTrees	500 trees; feature fraction 0.7; minimum leaf size 3; unlimited depth; squared-error criterion; bootstrap sampling disabled.
XGBoost	500 boosting rounds; depth 3; learning rate 0.03; row and column fractions 0.8; L_2 regularization 2.0; `reg:squarederror` objective.
LightGBM	500 boosting rounds; 15 leaves; learning rate 0.03; minimum child samples 15; column fraction 0.8; L_2 regularization 2.0; default regression objective; `subsample=0.8` with inactive row bagging.
CatBoost	500 iterations; depth 5; learning rate 0.03; leaf L_2 regularization 5.0; RMSE loss.

Later PH-VBP analyses evaluated HistGradientBoosting and CatBoost using the settings in Table M2.

Table S1. Complete continuous predictor list by view and engineered CMS source family.

Number	View	Source Family	Measure
1	healthcare	COMPLICATION	COMP_HIP_KNEE
2	healthcare	COMPLICATION	MORT_30_AMI
3	healthcare	COMPLICATION	MORT_30_CABG
4	healthcare	COMPLICATION	MORT_30_COPD
5	healthcare	COMPLICATION	MORT_30_HF
6	healthcare	COMPLICATION	MORT_30_PN
7	healthcare	COMPLICATION	MORT_30_STK
8	healthcare	COMPLICATION	PSI_03
9	healthcare	COMPLICATION	PSI_04
10	healthcare	COMPLICATION	PSI_06
11	healthcare	COMPLICATION	PSI_08
12	healthcare	COMPLICATION	PSI_09
13	healthcare	COMPLICATION	PSI_10
14	healthcare	COMPLICATION	PSI_11
15	healthcare	COMPLICATION	PSI_12
16	healthcare	COMPLICATION	PSI_13
17	healthcare	COMPLICATION	PSI_14
18	healthcare	COMPLICATION	PSI_15
19	healthcare	COMPLICATION	PSI_90
20	healthcare	HCAHPS	H_CLEAN_LINEAR_SCORE
21	healthcare	HCAHPS	H_COMP_1_LINEAR_SCORE
22	healthcare	HCAHPS	H_COMP_2_LINEAR_SCORE
23	healthcare	HCAHPS	H_COMP_3_LINEAR_SCORE
24	healthcare	HCAHPS	H_COMP_6_LINEAR_SCORE
25	healthcare	HCAHPS	H_COMP_7_LINEAR_SCORE
26	healthcare	HCAHPS	H_HSP_RATING_LINEAR_SCORE
27	healthcare	HCAHPS	H_QUIET_LINEAR_SCORE
28	healthcare	HCAHPS	H_RECMND_LINEAR_SCORE
29	healthcare	IMAGING	OP_10
30	healthcare	IMAGING	OP_13
31	healthcare	IMAGING	OP_39
32	healthcare	IMAGING	OP_8
33	healthcare	TIMELY	ED_2_STRATA_1
34	healthcare	TIMELY	ED_2_STRATA_2
35	healthcare	TIMELY	HCP_COVID_19
36	healthcare	TIMELY	IMM_3
37	healthcare	TIMELY	OP_18B
38	healthcare	TIMELY	OP_18C
39	healthcare	TIMELY	OP_2
40	healthcare	TIMELY	OP_22
41	healthcare	TIMELY	OP_23
42	healthcare	TIMELY	OP_29
43	healthcare	TIMELY	OP_31
44	healthcare	TIMELY	OP_3B
45	healthcare	TIMELY	SAFE_USE_OF_OPIOIDS
46	healthcare	TIMELY	SEP_1
47	healthcare	TIMELY	SEP_SH_3HR
48	healthcare	TIMELY	SEP_SH_6HR
49	healthcare	TIMELY	SEV_SEP_3HR
50	healthcare	TIMELY	SEV_SEP_6HR
51	healthcare	TIMELY	STK_02
52	healthcare	TIMELY	STK_03
53	healthcare	TIMELY	STK_05
54	healthcare	TIMELY	STK_06
55	healthcare	TIMELY	VTE_1
56	healthcare	TIMELY	VTE_2
57	healthcare	UNPLANNED_CMP	EDAC_30_AMI
58	healthcare	UNPLANNED_CMP	EDAC_30_HF
59	healthcare	UNPLANNED_CMP	EDAC_30_PN

Continued on next page

Number	View	Source Family	Measure
60	healthcare	UNPLANNED_CMP	OP_32
61	healthcare	UNPLANNED_CMP	OP_35_ADM
62	healthcare	UNPLANNED_CMP	OP_35_ED
63	healthcare	UNPLANNED_CMP	OP_36
64	healthcare	UNPLANNED_CMP	READM_30_AMI
65	healthcare	UNPLANNED_CMP	READM_30_CABG
66	healthcare	UNPLANNED_CMP	READM_30_COPD
67	healthcare	UNPLANNED_CMP	READM_30_HF
68	healthcare	UNPLANNED_CMP	READM_30_HIP_KNEE
69	healthcare	UNPLANNED_CMP	READM_30_HOSP_WIDE
70	healthcare	UNPLANNED_CMP	READM_30_PN
71	healthcare	UNPLANNED	EDAC_30_AMI
72	healthcare	UNPLANNED	EDAC_30_HF
73	healthcare	UNPLANNED	EDAC_30_PN
74	healthcare	UNPLANNED	OP_32
75	healthcare	UNPLANNED	OP_35_ADM
76	healthcare	UNPLANNED	OP_35_ED
77	healthcare	UNPLANNED	OP_36
78	healthcare	UNPLANNED	READM_30_AMI
79	healthcare	UNPLANNED	READM_30_CABG
80	healthcare	UNPLANNED	READM_30_COPD
81	healthcare	UNPLANNED	READM_30_HF
82	healthcare	UNPLANNED	READM_30_HIP_KNEE
83	healthcare	UNPLANNED	READM_30_HOSP_WIDE
84	healthcare	UNPLANNED	READM_30_PN
85	insurance	MSPB	MSPB_1
86	insurance	PAYMENT_CMP	PAYM_30_AMI
87	insurance	PAYMENT_CMP	PAYM_30_HF
88	insurance	PAYMENT_CMP	PAYM_30_PN
89	insurance	PAYMENT_CMP	PAYM_90_HIP_KNEE
90	insurance	PAYMENT	PAYM_30_AMI
91	insurance	PAYMENT	PAYM_30_HF
92	insurance	PAYMENT	PAYM_30_PN
93	insurance	PAYMENT	PAYM_90_HIP_KNEE
94	safety_medication_proxy	HAI_CMP	HAI_1_SIR
95	safety_medication_proxy	HAI_CMP	HAI_2_SIR
96	safety_medication_proxy	HAI_CMP	HAI_3_SIR
97	safety_medication_proxy	HAI_CMP	HAI_4_SIR
98	safety_medication_proxy	HAI_CMP	HAI_5_SIR
99	safety_medication_proxy	HAI_CMP	HAI_6_SIR
100	safety_medication_proxy	HAI_SIR	HAI_1_SIR
101	safety_medication_proxy	HAI_SIR	HAI_2_SIR
102	safety_medication_proxy	HAI_SIR	HAI_3_SIR
103	safety_medication_proxy	HAI_SIR	HAI_4_SIR
104	safety_medication_proxy	HAI_SIR	HAI_5_SIR
105	safety_medication_proxy	HAI_SIR	HAI_6_SIR
106	safety_medication_proxy	HCAHPS	H_COMP_5_LINEAR_SCORE

Table S2. Complete constrained-feature specification, transformation, expected sign, and rationale.

Feature	View	Transformation	Expected Sign	Reason
H__TIMELY__ED_2_STRATA_1	healthcare	published score; adverse waiting/time measures multiplied by -1	positive	Shorter waits and lower adverse rates are favorable
H__TIMELY__ED_2_STRATA_2	healthcare	published score; adverse waiting/time measures multiplied by -1	positive	Shorter waits and lower adverse rates are favorable
H__TIMELY__OP_18B	healthcare	published score; adverse waiting/time measures multiplied by -1	positive	Shorter waits and lower adverse rates are favorable
H__TIMELY__OP_18C	healthcare	published score; adverse waiting/time measures multiplied by -1	positive	Shorter waits and lower adverse rates are favorable
H__TIMELY__OP_22	healthcare	published score; adverse waiting/time measures multiplied by -1	positive	Shorter waits and lower adverse rates are favorable
H__UNPLANNED__EDAC_30_AMI	healthcare	negative of the published score	positive	Lower unplanned use is favorable
H__UNPLANNED__EDAC_30_HF	healthcare	negative of the published score	positive	Lower unplanned use is favorable
H__UNPLANNED__EDAC_30_PN	healthcare	negative of the published score	positive	Lower unplanned use is favorable
H__UNPLANNED__OP_32	healthcare	negative of the published score	positive	Lower unplanned use is favorable
H__UNPLANNED__OP_35_ADM	healthcare	negative of the published score	positive	Lower unplanned use is favorable
H__UNPLANNED__OP_35_ED	healthcare	negative of the published score	positive	Lower unplanned use is favorable
H__UNPLANNED__OP_36	healthcare	negative of the published score	positive	Lower unplanned use is favorable
H__UNPLANNED__READM_30_AMI	healthcare	negative of the published score	positive	Lower unplanned use is favorable
H__UNPLANNED__READM_30_CABG	healthcare	negative of the published score	positive	Lower unplanned use is favorable
H__UNPLANNED__READM_30_COPD	healthcare	negative of the published score	positive	Lower unplanned use is favorable
H__UNPLANNED__READM_30_HF	healthcare	negative of the published score	positive	Lower unplanned use is favorable
H__UNPLANNED__READM_30_HIP_KNEE	healthcare	negative of the published score	positive	Lower unplanned use is favorable
H__UNPLANNED__READM_30_HOSP_WIDE	healthcare	negative of the published score	positive	Lower unplanned use is favorable
H__UNPLANNED__READM_30_PN	healthcare	negative of the published score	positive	Lower unplanned use is favorable
I__MSPB__MSPB_1	insurance	negative of the published MSPB score	positive	Lower spending is favorable
P__HAI_SIR__HAI_1_SIR	safety_medication_proxy	-log(max(published SIR, 1e-4))	positive	Lower infection SIR is favorable
P__HAI_SIR__HAI_2_SIR	safety_medication_proxy	-log(max(published SIR, 1e-4))	positive	Lower infection SIR is favorable
P__HAI_SIR__HAI_3_SIR	safety_medication_proxy	-log(max(published SIR, 1e-4))	positive	Lower infection SIR is favorable
P__HAI_SIR__HAI_4_SIR	safety_medication_proxy	-log(max(published SIR, 1e-4))	positive	Lower infection SIR is favorable
P__HAI_SIR__HAI_5_SIR	safety_medication_proxy	-log(max(published SIR, 1e-4))	positive	Lower infection SIR is favorable
P__HAI_SIR__HAI_6_SIR	safety_medication_proxy	-log(max(published SIR, 1e-4))	positive	Lower infection SIR is favorable
P__HCAHPS__H_COMP_5_LINEAR_SCORE	safety_medication_proxy	published HCAHPS linear mean score	positive	Higher patient-reported score is favorable

Supplementary release, outcome, and validation audits

The PH-Star extension retained release-level prediction because the overall star rating can update between snapshots. PH-VBP was reconstructed at the fiscal-year level after auditing the source panel: all 624 hospital–fiscal-year groups represented more than once had one unique TPS within the group. Consequently, no repeated annual TPS row was treated as an independent outcome update.

Table S3. Release-aware cohorts used for model development, selection, and temporal validation.

Outcome	Role	Feature release	Target release	n	Hospitals	Tier 1/2/3/4/5
PH-Star	Development train	2023-10-06 to 2024-04-24	next snapshot	945	325	109/263/304/173/96
PH-Star	Validation	2024-07-31	2024-10-30	301	301	36/90/86/65/24
PH-Star	Original temporal test	2024-10-30 and 2025-02-19	next snapshot	603	302	72/176/173/134/48
PH-Star	Post-development	2025-04-30 to 2026-02-25	next snapshot	1,236	338	140/343/400/268/85
PH-VBP	FY2024 development	2023-10-06	2024-01-31	321	321	64/65/62/65/65
PH-VBP	FY2025 tuning	2024-10-30	2025-02-19	301	301	59/47/71/54/70
PH-VBP	Locked historical refit	FY2024 and FY2025 pairs	–	622	338	123/112/133/119/135
PH-VBP	FY2026 temporal validation	2025-11-26	2026-02-25	308	308	6/32/48/74/148

Table S3b. Hospital-ID overlap matrices for the original PH-Star splits and annual PH-VBP phases. Diagonal entries are unique hospitals within each phase.

Outcome	Row phase	Development/train	Validation/tuning	Temporal test/validation
PH-Star	Train	325	300	286
PH-Star	Validation	300	301	287
PH-Star	Temporal test	286	287	302
PH-VBP	FY2024 development	321	284	282
PH-VBP	FY2025 tuning	284	301	290
PH-VBP	FY2026 validation	282	290	308

The locked PH-VBP cutoffs were 12.75, 17.50, 22.9167, and 29.50. Scores exactly equal to a cutoff were assigned to the lower tier; FY2024 contained 2, 2, 1, and 2 exact ties at the respective boundaries. The annual TPS means were 21.558, 22.509, and 29.838 in FY2024, FY2025, and FY2026.

Table S4a. PH-Star relation-weighting sensitivity on the original validation split. The prespecified current-$k = 5$ graph was retained because the adaptive-$k = 8$ QWK difference was 0.0001.

Configuration	QWK	Macro-F1	$\mathrm{MAE_T}$	Low-tier recall
adaptive_k8	0.829 ± 0.004	0.537	0.488	0.624
current_k5	0.829 ± 0.005	0.532	0.494	0.636
neighbor_global_k5	0.800 ± 0.058	0.474	0.568	0.604

Table S4b. FY2025 annual PH-VBP architecture and view ablations (301 hospitals). Values are mean ± SD across five fits. Selection used QWK. Table S11c reports paired confidence intervals from the five-seed ensembles.

Variant	QWK	Macro-F1	MAE_T	Low-tier recall
selected_no_graph_tps	0.716 ± 0.023	0.413 ± 0.008	0.743 ± 0.035	0.456 ± 0.055
no_monotonic	0.715 ± 0.026	0.441 ± 0.025	0.728 ± 0.047	0.518 ± 0.053
adaptive_graph_k8_tps	0.710 ± 0.018	0.419 ± 0.010	0.753 ± 0.023	0.497 ± 0.017
no_healthcare_view	0.702 ± 0.010	0.424 ± 0.010	0.756 ± 0.017	0.444 ± 0.034
no_insurance_view	0.679 ± 0.018	0.411 ± 0.008	0.779 ± 0.016	0.446 ± 0.057
current_graph_k5_tps	0.678 ± 0.041	0.407 ± 0.022	0.766 ± 0.021	0.441 ± 0.080
ordinal_head_only	0.642 ± 0.028	0.288 ± 0.034	0.966 ± 0.073	0.460 ± 0.016
no_safety_proxy_view	0.581 ± 0.030	0.340 ± 0.014	0.902 ± 0.015	0.355 ± 0.045

Table S4c. Continuous-TPS regression comparators on FY2025 tuning data; all predictions use the same locked cutoffs.

Method	QWK	Macro-F1	MAE_T	TPS MAE	TPS RMSE
hist_gradient_boosting	0.697	0.443	0.704	5.360	7.207
xgboost	0.670	0.424	0.738	5.339	7.144
catboost	0.662	0.385	0.756	5.390	7.070
lightgbm	0.660	0.414	0.751	5.508	7.286
extra_trees	0.638	0.350	0.775	5.657	7.350
random_forest	0.592	0.317	0.811	5.838	7.518

Table S4d. Five-seed computational profile on the stated four-core/eight-thread CPU environment. Times are seconds and memory is peak process RSS in MB.

Outcome	Parameters	Training	Inference	Peak memory	Model MB
PH-Star	282,207	70.2 ± 3.5	0.0087 ± 0.0010	611 ± 5	1.09
PH-VBP FY2024 selection fit	280,418	3.53 ± 0.46	0.0048 ± 0.0004	412 ± 3	1.09

Table S5. Final task-specific performance and PH-VBP refit sensitivity. Parentheses for ensemble QWK are 95% hospital-block intervals.

Outcome/period	Estimate	n	QWK	Macro-F1	MAE_T	Low-tier recall
PH-Star post-development	Five-fit mean	1236	0.757 ± 0.013	0.509 ± 0.013	0.524 ± 0.012	0.513 ± 0.083
PH-Star original test	Five-fit mean	603	0.816 ± 0.009	0.584 ± 0.036	0.455 ± 0.037	0.600 ± 0.015
PH-Star original test	Five-seed ensemble	603	0.824 (0.792–0.851)	0.578	0.453	0.602
PH-Star post-development	Five-seed ensemble	1236	0.768 (0.733–0.798)	0.512	0.513	0.519
PH-VBP FY2026, FY2024-only fit	Five-fit mean	308	0.573 ± 0.051	0.338 ± 0.026	0.807 ± 0.090	0.450 ± 0.048
PH-VBP FY2025 tuning	Five-fit mean	301	0.716 ± 0.023	0.413 ± 0.008	0.743 ± 0.035	0.456 ± 0.055
PH-VBP FY2025 tuning	Five-seed ensemble	301	0.759 (0.713–0.804)	0.436	0.681	0.459
PH-VBP FY2026, FY2024-only fit	Five-seed ensemble	308	0.607 (0.537–0.672)	0.362	0.750	0.479
PH-VBP FY2026, pooled locked refit	Five-fit mean	308	0.678 ± 0.021	0.392 ± 0.034	0.636 ± 0.059	0.490 ± 0.115
PH-VBP FY2026, pooled locked refit	Five-seed ensemble	308	0.717 (0.657–0.773)	0.402	0.584	0.469
PH-VBP FY2026, hospital-disjoint	Five-fit mean	308	0.638 ± 0.051	0.399 ± 0.025	0.619 ± 0.051	0.315 ± 0.055
PH-VBP FY2026, hospital-disjoint	Five-seed ensemble	308	0.689 (0.618–0.747)	0.427	0.545	0.286
PH-VBP FY2026, region-disjoint	Five-fit mean	308	0.545 ± 0.025	0.323 ± 0.027	0.742 ± 0.097	0.205 ± 0.078
PH-VBP FY2026, region-disjoint	Five-seed ensemble	308	0.636 (0.562–0.699)	0.378	0.636	0.240

Table S6a. Conventional comparators in post-development, locked-refit, and separated-group temporal validation. PH-VBP rows are five-seed TPS ensembles.

Outcome	Method	n	QWK	Macro-F1	MAE_T	Low-tier recall
PH-Star post-development	catboost	1236	0.705 ± 0.009	0.477 ± 0.006	0.511 ± 0.009	0.281
PH-Star post-development	random_forest	1236	0.680 ± 0.005	0.455 ± 0.007	0.527 ± 0.005	0.289
PH-VBP pooled refit	CatBoost	308	0.639	0.384	0.711	0.469
PH-VBP pooled refit	HIOP-GL	308	0.717	0.402	0.584	0.469
PH-VBP pooled refit	HistGradientBoosting	308	0.630	0.409	0.714	0.568
PH-VBP hospital disjoint	CatBoost	308	0.630	0.409	0.705	0.500
PH-VBP hospital disjoint	HIOP-GL	308	0.689	0.427	0.545	0.286
PH-VBP hospital disjoint	HistGradientBoosting	308	0.629	0.384	0.698	0.401
PH-VBP region disjoint	CatBoost	308	0.562	0.351	0.828	0.500
PH-VBP region disjoint	HIOP-GL	308	0.636	0.378	0.636	0.240
PH-VBP region disjoint	HistGradientBoosting	308	0.524	0.359	0.838	0.453

Note to Table S5: PH-Star analysis versions. For PH-Star, the five-fit summaries and five-seed ensembles in Table S5 are from the final temporal-validation analysis. The original-test cohort comprises 603 hospital-release observations. The HIOP-GL diagnostics in Tables S9a and S10a use the earlier benchmark fits on the same cohort and are reported separately. These are different fitted model sets, not independent test cohorts.

Table S6b. Paired post-development hospital-block comparisons. Positive deltas favor HIOP for QWK, Macro-F1, and recall; negative deltas favor HIOP for $\mathrm{MAE_T}$. Holm adjustment is within outcome or validation design and metric.

Outcome	Comparison	Metric	Delta	Lower	Upper	Holm p
PH-Star post-development	HIOP minus random_forest	macro_f1	0.044	-0.009	0.097	0.216
PH-Star post-development	HIOP minus random_forest	qwk	0.072	0.038	0.108	0.002
PH-Star post-development	HIOP minus random_forest	mae_t	0.007	-0.044	0.055	1.000
PH-Star post-development	HIOP minus random_forest	low_tier_recall	0.224	0.153	0.296	0.002
PH-Star post-development	HIOP minus catboost	macro_f1	0.033	-0.014	0.083	0.216
PH-Star post-development	HIOP minus catboost	qwk	0.057	0.024	0.091	0.002
PH-Star post-development	HIOP minus catboost	mae_t	0.012	-0.039	0.062	1.000
PH-Star post-development	HIOP minus catboost	low_tier_recall	0.242	0.171	0.312	0.002
PH-VBP pooled refit	HIOP minus HistGradientBoosting	macro_f1	-0.007	-0.075	0.054	1.000
PH-VBP pooled refit	HIOP minus HistGradientBoosting	qwk	0.087	0.043	0.134	0.002
PH-VBP pooled refit	HIOP minus HistGradientBoosting	mae_t	-0.129	-0.211	-0.045	0.006
PH-VBP pooled refit	HIOP minus HistGradientBoosting	brier_avg	-0.035	-0.061	-0.009	0.010
PH-VBP pooled refit	HIOP minus HistGradientBoosting	low_tier_recall	-0.098	-0.300	0.037	0.598
PH-VBP pooled refit	HIOP minus CatBoost	macro_f1	0.019	-0.054	0.089	1.000
PH-VBP pooled refit	HIOP minus CatBoost	qwk	0.077	0.033	0.123	0.002
PH-VBP pooled refit	HIOP minus CatBoost	mae_t	-0.125	-0.205	-0.049	0.006
PH-VBP pooled refit	HIOP minus CatBoost	brier_avg	-0.044	-0.069	-0.018	0.008
PH-VBP pooled refit	HIOP minus CatBoost	low_tier_recall	0.005	-0.267	0.283	1.000
PH-VBP hospital disjoint	HIOP minus HistGradientBoosting	macro_f1	0.041	-0.071	0.158	0.972
PH-VBP hospital disjoint	HIOP minus HistGradientBoosting	qwk	0.060	-0.002	0.121	0.072
PH-VBP hospital disjoint	HIOP minus HistGradientBoosting	mae_t	-0.153	-0.244	-0.068	0.000
PH-VBP hospital disjoint	HIOP minus HistGradientBoosting	brier_avg	-0.047	-0.074	-0.018	0.001
PH-VBP hospital disjoint	HIOP minus HistGradientBoosting	low_tier_recall	-0.119	-0.452	0.195	0.458
PH-VBP hospital disjoint	HIOP minus CatBoost	macro_f1	0.017	-0.083	0.110	0.972
PH-VBP hospital disjoint	HIOP minus CatBoost	qwk	0.058	0.003	0.116	0.072
PH-VBP hospital disjoint	HIOP minus CatBoost	mae_t	-0.159	-0.247	-0.071	0.001
PH-VBP hospital disjoint	HIOP minus CatBoost	brier_avg	-0.057	-0.083	-0.029	0.000
PH-VBP hospital disjoint	HIOP minus CatBoost	low_tier_recall	-0.219	-0.483	0.000	0.108
PH-VBP region disjoint	HIOP minus HistGradientBoosting	macro_f1	0.015	-0.074	0.108	1.000
PH-VBP region disjoint	HIOP minus HistGradientBoosting	qwk	0.111	0.041	0.178	0.002
PH-VBP region disjoint	HIOP minus HistGradientBoosting	mae_t	-0.200	-0.302	-0.104	0.000
PH-VBP region disjoint	HIOP minus HistGradientBoosting	brier_avg	-0.022	-0.049	0.005	0.126
PH-VBP region disjoint	HIOP minus HistGradientBoosting	low_tier_recall	-0.214	-0.480	0.013	0.081
PH-VBP region disjoint	HIOP minus CatBoost	macro_f1	0.024	-0.072	0.121	1.000
PH-VBP region disjoint	HIOP minus CatBoost	qwk	0.072	0.008	0.132	0.032
PH-VBP region disjoint	HIOP minus CatBoost	mae_t	-0.191	-0.276	-0.097	0.000
PH-VBP region disjoint	HIOP minus CatBoost	brier_avg	-0.039	-0.066	-0.012	0.016
PH-VBP region disjoint	HIOP minus CatBoost	low_tier_recall	-0.262	-0.525	-0.029	0.064

Table S7a. Prespecified data-integrity checks.

Dataset	Check	Observed	Expected	Status
development_panel	unique_hospital_release_rows	0	0	pass
development_panel	target_after_feature_release	0	0	pass
development_panel	valid_ph_star_tier	1849	1849	pass
development_panel	valid_ph_vbp_tier	1849	1849	pass
post_development_panel	unique_hospital_release_rows	0	0	pass
post_development_panel	target_after_feature_release	0	0	pass
post_development_panel	valid_ph_star_tier	1236	1236	pass
post_development_panel	valid_ph_vbp_tier	1236	1236	pass

Table S7b. PH-VBP target drift under the annual design.

Phase	Rows	Hospitals	FY	Mean	SD	Median	Q1	Q3
development_fy2024	321	321	2024	21.558	9.783	20.583	14.583	27.000
external_fy2026	308	308	2026	29.838	10.628	28.750	22.062	35.688
tuning_fy2025	301	301	2025	22.509	10.610	21.000	14.083	28.667

Table S7c. Post-development schema coverage by release and predictor view.

Feature release	View	Development columns	Observed columns	Coverage	Cell missing rate
2025-04-30	healthcare	87	85	0.977	0.258
2025-04-30	insurance	9	9	1.000	0.198
2025-04-30	safety_medication_proxy	13	13	1.000	0.306
2025-08-14	healthcare	87	83	0.954	0.285
2025-08-14	insurance	9	1	0.111	0.889
2025-08-14	safety_medication_proxy	13	13	1.000	0.309
2025-11-26	healthcare	87	77	0.885	0.299
2025-11-26	insurance	9	1	0.111	0.889
2025-11-26	safety_medication_proxy	13	13	1.000	0.299
2026-02-25	healthcare	87	75	0.862	0.339
2026-02-25	insurance	9	1	0.111	0.889
2026-02-25	safety_medication_proxy	13	13	1.000	0.330

Table S7d. FY2026 PH-VBP per-tier performance after locked historical refitting for HIOP-GL and the strongest ensemble-QWK conventional comparator.

Method	Tier	Support	Predicted	Precision	Recall	Adjacent error	Distant error
HIOP-GL	1	6	18	0.167	0.500	0.500	0.000
HIOP-GL	2	32	50	0.280	0.438	0.469	0.094
HIOP-GL	3	48	39	0.282	0.229	0.667	0.104
HIOP-GL	4	74	84	0.381	0.432	0.419	0.149
HIOP-GL	5	148	117	0.863	0.682	0.257	0.061
CatBoost	1	6	12	0.250	0.500	0.500	0.000
CatBoost	2	32	46	0.304	0.438	0.531	0.031
CatBoost	3	48	86	0.267	0.479	0.500	0.021
CatBoost	4	74	103	0.282	0.392	0.473	0.135
CatBoost	5	148	61	0.951	0.392	0.453	0.155

Table S7e. FY2026 locked-refit PH-VBP confusion matrices. Rows are true tiers and columns are predicted tiers.

Method	True tier	Pred. 1	Pred. 2	Pred. 3	Pred. 4	Pred. 5
HIOP-GL	1	3	3	0	0	0
HIOP-GL	2	9	14	6	3	0
HIOP-GL	3	4	21	11	11	1
HIOP-GL	4	2	9	16	32	15
HIOP-GL	5	0	3	6	38	101
CatBoost	1	3	3	0	0	0
CatBoost	2	7	14	10	1	0
CatBoost	3	1	18	23	6	0
CatBoost	4	1	9	32	29	3
CatBoost	5	0	2	21	67	58

Table S7f. PH-VBP separated-group temporal-validation partitions. Counts are identical across seeds. Hospital overlap is zero in both designs; state overlap is additionally zero in the region-disjoint design.

Design	Partition	Train rows	Train hospitals	FY2026 rows	FY2026 hospitals	Hospital overlap	State overlap
hospital disjoint	fold_1	509	277	56	56	0	25
hospital disjoint	fold_2	493	268	63	63	0	20
hospital disjoint	fold_3	492	271	62	62	0	22
hospital disjoint	fold_4	494	267	65	65	0	21
hospital disjoint	fold_5	500	269	62	62	0	26
region disjoint	northeast	583	315	18	18	0	0
region disjoint	midwest	501	275	57	57	0	0
region disjoint	south	288	155	167	167	0	0
region disjoint	west	494	269	66	66	0	0

Table S7g. HIOP-GL performance within each held-out Census region. Unequal regional sample sizes make these transport diagnostics rather than evidence of performance equivalence.

Held-out region	n	QWK	Macro-F1	$\mathrm{MAE_T}$	Low-tier recall
midwest	57	0.564	0.256	0.649	0.000
northeast	18	0.706	0.362	0.667	0.300
south	167	0.624	0.377	0.671	0.314
west	66	0.643	0.395	0.530	0.125

Supplementary ownership transport and independent-authority criterion checks

After all PH-Star model choices and weights were frozen, three supplementary checks were conducted without refitting. They answer non-equivalent questions and are reported separately rather than combined as independent same-outcome validation. The first tests ownership transport while retaining the CMS feature and outcome system. The five-seed ensemble was applied to the 6 October 2023 CMS feature release for 5,446 US hospitals. The external cohort comprised 302 California non-public acute-care hospitals; the public-ownership filter used for every development phase excluded all of these facilities, and their facility-ID overlap with the development, validation, and temporal-test panel was zero. Graph relations were reconstructed from predictor and facility information within the scoring release without using external outcomes. Linking the 6 October 2023 features to the 31 January 2024 PH-Star retained 229 hospitals with a reported rating. Ten thousand hospital bootstrap resamples generated percentile intervals.

The second check tests criterion concordance with the California Department of Health Care Access and Information (HCAI), an independent reporting authority, rather than reproduction of CMS PH-Star. CMS Certification Numbers were linked to HCAI identifiers only through the official Licensed and Certified Healthcare Facility Crosswalk. The broad criterion averaged all available 2023 HCAI performance ratings after coding Better as 1, As Expected as 0, and Worse as -1; hospitals required at least five ratings. Ratings covered inpatient mortality, postoperative sepsis, pediatric indicators, CABG, elective PCI, and TAVR where reportable. A mortality-specific sensitivity used 2024 HCAI risk-adjusted mortality rates for acute myocardial infarction, acute stroke, gastrointestinal hemorrhage, heart failure, hip fracture, and pneumonia. Within-condition percentiles were averaged after reversing direction, with at least four conditions required.

The third check uses 2024 hospital-level Inpatient Quality Indicator composites maintained by the New York State Department of Health (NYSDOH) from all-payer SPARCS discharges. Non-public New York hospitals were scored from frozen 6 October 2023 features. The primary linkage required exact normalized-name agreement, county agreement, and a unique one-to-one match; ambiguous campus and health-system links were rejected. Both IQI90 (mortality for selected procedures) and IQI91 (mortality for selected conditions) were required. The external quality criterion was the negative mean log of the two risk-adjusted composite measures, so larger values represented lower mortality. An expanded sensitivity admitted unique high-confidence same-county name matches with similarity at least 0.86 and a margin of at least 0.08 over the next candidate. Linkage representativeness was audited by comparing the frozen-score distribution and hospital-type and ownership composition of exact-linked and unmatched eligible hospitals. Correlation intervals used 10,000 hospital bootstrap resamples and two-sided permutation tests used 99,999 shuffles. The California broad criterion, California mortality sensitivity, and primary New York composite were Holm-adjusted together. A more conservative sensitivity Holm-adjusted all six model-to-external-criterion associations, including expanded linkage and the two New York components. These are independent-authority criterion checks, not same-outcome PH-Star validation or prospective deployment. Source data are available at `https://data.chhs.ca.gov/dataset/california-hospital-performance-ratings`, `https://data.chhs.ca.gov/dataset/california-hospital-inpatient-mortality-rates-and-quality-ratings`, `https://data.chhs.ca.gov/dataset/licensed-facility-crosswalk`, and `https://health.data.ny.gov/d/ba3n-bkk4`.

Table S8a. Frozen PH-Star ensemble performance in the ownership-external California non-public hospital cohort. Intervals are 95% hospital-bootstrap percentile intervals.

Metric	n	Estimate	Lower	Upper
QWK	229	0.637	0.546	0.718
Macro-F1	229	0.441	0.377	0.500
Tier-distance MAE	229	0.721	0.616	0.825
Low-tier recall	229	0.387	0.259	0.517
Accuracy	229	0.467	0.402	0.533
Class-averaged Brier	229	0.141	0.131	0.152
ECE (10 bins)	229	0.159	0.103	0.226

Table S8b. Independent-authority criterion checks of the frozen expected PH-Star tier across California and New York. Positive Spearman ρ indicates concordance with better independently reported performance. Holm adjustment covers all three rows; these are not same-outcome PH-Star validations.

External criterion	n	ρ	Lower	Upper	Permutation p	Holm p
2023 California HCAI multi-measure performance	243	0.247	0.125	0.366	0.0002	0.0006
2024 California HCAI mortality sensitivity	233	0.096	-0.035	0.229	0.14356	0.14356
2024 NYSDOH IQI90/IQI91 mortality composite	57	0.451	0.195	0.663	0.00048	0.00096

Table S8c. Coverage-threshold sensitivity for the 2023 HCAI multi-measure performance criterion.

Minimum rated measures	n	ρ
3	249	0.241
5	243	0.247
8	201	0.273
10	162	0.279
12	131	0.334

Table S8d. Conservative NYSDOH-to-CMS hospital linkage flow. Ambiguous, non-unique, campus-level, and low-confidence links were excluded.

Linkage stage	n
NYSDOH hospitals with both 2024 IQI composites	185
Non-public New York hospitals with frozen model scores	142
Unique exact normalized-name and county matches (primary)	57
Unique exact or high-confidence matches (expanded sensitivity)	67

Table S8e. NYSDOH criterion and linkage sensitivities. The primary model analysis uses only exact normalized-name and county matches; component and expanded-link results are sensitivity analyses.

Analysis	n	ρ	Lower	Upper	Permutation p
Joint IQI90/IQI91 criterion, exact links	57	0.451	0.195	0.663	0.00048
Joint criterion, expanded links	67	0.415	0.161	0.628	0.0006
IQI90 selected-procedure mortality	57	0.179	-0.138	0.464	0.18342
IQI91 selected-condition mortality	57	0.441	0.192	0.647	0.0007
Observed next-release PH-Star reference	46	0.529	0.275	0.724	0.00021

Table S8f. Linkage-representativeness audit for the frozen expected PH-Star tier. The exact-linked versus other-eligible standardized mean difference was 0.066; Mann–Whitney $p = 0.464$ and Kolmogorov–Smirnov $p = 0.545$.

Linkage group	n	Mean	SD	Median	Q1	Q3
Exact linked	57	2.554	1.023	2.623	1.655	3.114
Other eligible	85	2.479	1.202	2.258	1.495	3.110

Table S8g. Hospital-type and ownership composition of exact-linked and other eligible New York hospitals. Similar frozen-score distributions do not remove composition-related transportability limits.

Characteristic	Category	Exact linked, n (%)	Other eligible, n (%)
Hospital type	Acute Care Hospitals	45 (78.9%)	73 (85.9%)
Hospital type	Childrens	0 (0.0%)	1 (1.2%)
Hospital type	Critical Access Hospitals	12 (21.1%)	5 (5.9%)
Hospital type	Psychiatric	0 (0.0%)	6 (7.1%)
Ownership	Proprietary	1 (1.8%)	5 (5.9%)
Ownership	Voluntary non-profit - Church	6 (10.5%)	6 (7.1%)
Ownership	Voluntary non-profit - Other	4 (7.0%)	6 (7.1%)
Ownership	Voluntary non-profit - Private	46 (80.7%)	68 (80.0%)

Table S8h. Conservative familywise sensitivity across all six model-to-external-criterion associations. The observed next-release PH-Star reference is descriptive and is not included.

External association	Role	Raw p	Six-association Holm p
2023 California HCAI multi-measure performance	primary external criterion	0.0002	0.0012
2024 California HCAI mortality	criterion sensitivity	0.14356	0.28712
2024 NYSDOH joint IQI90/IQI91 exact links	primary external criterion	0.00048	0.0024
2024 NYSDOH joint IQI90/IQI91 expanded links	linkage sensitivity	0.0006	0.0024
2024 NYSDOH IQI90 selected-procedure mortality	component sensitivity	0.18342	0.28712
2024 NYSDOH IQI91 selected-condition mortality	component sensitivity	0.0007	0.0024

Repeated-outcome audit. The development panel contained 624 hospital–fiscal-year groups represented in more than one release row. All 624 had exactly one unique TPS, and all were marked as repeated identical outcomes. This audit motivated the one-pair-per-fiscal-year PH-VBP design.

Supplementary PH-Star benchmark and diagnostic detail

The following tables retain the prespecified PH-Star temporal-test analyses. PH-VBP rows from the earlier quarterly formulation are intentionally not carried forward because annual TPS did not update independently within fiscal years.

Table S9a. Original PH-Star temporal-test per-tier results (603 hospital-release observations). Precision, recall, adjacent-tier error, and distant-tier error are reported for selected methods.

Target	Method	Tier	Support	Precision	Recall	Adjacent Error Rate	Distant Error Rate
PH-Star	Random forest	1	72	1.0000	0.2500	0.6944	0.0556
PH-Star	Random forest	2	176	0.6439	0.7500	0.2443	0.0057
PH-Star	Random forest	3	173	0.6611	0.9133	0.0867	0.0000
PH-Star	Random forest	4	134	0.8981	0.7239	0.2090	0.0672
PH-Star	Random forest	5	48	0.9697	0.6667	0.1667	0.1667
PH-Star	CatBoost	1	72	0.9333	0.1944	0.7222	0.0833
PH-Star	CatBoost	2	176	0.5678	0.6420	0.3239	0.0341
PH-Star	CatBoost	3	173	0.6144	0.8382	0.1561	0.0058
PH-Star	CatBoost	4	134	0.7934	0.7164	0.2015	0.0821
PH-Star	CatBoost	5	48	0.9062	0.6042	0.2708	0.1250
PH-Star	HIOP-GL	1	72	0.5283	0.7778	0.1528	0.0694
PH-Star	HIOP-GL	2	176	0.6549	0.4205	0.5739	0.0057
PH-Star	HIOP-GL	3	173	0.5590	0.7399	0.2197	0.0405
PH-Star	HIOP-GL	4	134	0.7826	0.4030	0.5821	0.0149
PH-Star	HIOP-GL	5	48	0.5000	0.8958	0.0417	0.0625
PH-Star	TabPFN v1	1	72	0.5682	0.6944	0.3056	0.0000
PH-Star	TabPFN v1	2	176	0.5025	0.5682	0.4205	0.0114
PH-Star	TabPFN v1	3	173	0.5353	0.5260	0.4740	0.0000
PH-Star	TabPFN v1	4	134	0.7030	0.5299	0.3955	0.0746
PH-Star	TabPFN v1	5	48	0.7111	0.6667	0.2500	0.0833

Analysis-version note: earlier PH-Star benchmark fits. The HIOP-GL entries in Table S9a use the earlier benchmark fits on the original 603-observation temporal-test cohort. They are distinct from the final temporal-validation fits summarized in Table 3 and Table S5. Within each analysis, probabilities were averaged across five fits before assigning tiers. The test cohort is the same; the fitted models differ.

Table S9b. Post-development PH-Star per-tier performance of five-seed ensembles ($n = 1{,}236$ hospital-release observations; 338 hospitals). Original temporal-test results appear in Table S9a.

Model	True tier	Support	Precision	Recall	F1
HIOP-GL	1	140	0.610	0.614	0.612
HIOP-GL	2	343	0.604	0.423	0.497
HIOP-GL	3	400	0.497	0.677	0.574
HIOP-GL	4	268	0.490	0.287	0.362
HIOP-GL	5	85	0.399	0.718	0.513
CatBoost	1	140	0.824	0.100	0.178
CatBoost	2	343	0.508	0.452	0.478
CatBoost	3	400	0.503	0.730	0.595
CatBoost	4	268	0.573	0.571	0.572
CatBoost	5	85	0.652	0.506	0.570
Random forest	1	140	0.895	0.121	0.214
Random forest	2	343	0.521	0.466	0.492
Random forest	3	400	0.480	0.790	0.597
Random forest	4	268	0.594	0.425	0.496
Random forest	5	85	0.661	0.459	0.542

Each model averages its five seed-specific probability vectors and then applies argmax (lowest tier on an exact tie). Supports are true-class counts. Precision, recall and F1 are reported on a 0–1 scale, with zero-division values set to zero. The five supports sum to 1,236 for each model; observations from the same hospital may recur across releases.

Table S10a. Original PH-Star temporal-test confusion matrices (603 hospital-release observations). Rows are true tiers and columns are predicted tiers.

Target	Method	True Tier	Pred 1	Pred 2	Pred 3	Pred 4	Pred 5
PH-Star	CatBoost	1	14.0000	52.0000	4.0000	2.0000	0.0000
PH-Star	CatBoost	2	1.0000	113.0000	56.0000	6.0000	0.0000
PH-Star	CatBoost	3	0.0000	23.0000	145.0000	4.0000	1.0000
PH-Star	CatBoost	4	0.0000	11.0000	25.0000	96.0000	2.0000
PH-Star	CatBoost	5	0.0000	0.0000	6.0000	13.0000	29.0000
PH-Star	HIOP-GL	1	56.0000	11.0000	5.0000	0.0000	0.0000
PH-Star	HIOP-GL	2	48.0000	74.0000	53.0000	1.0000	0.0000
PH-Star	HIOP-GL	3	2.0000	26.0000	128.0000	12.0000	5.0000
PH-Star	HIOP-GL	4	0.0000	2.0000	40.0000	54.0000	38.0000
PH-Star	HIOP-GL	5	0.0000	0.0000	3.0000	2.0000	43.0000
PH-Star	Random forest	1	18.0000	50.0000	3.0000	1.0000	0.0000
PH-Star	Random forest	2	0.0000	132.0000	43.0000	1.0000	0.0000
PH-Star	Random forest	3	0.0000	14.0000	158.0000	1.0000	0.0000
PH-Star	Random forest	4	0.0000	9.0000	27.0000	97.0000	1.0000
PH-Star	Random forest	5	0.0000	0.0000	8.0000	8.0000	32.0000
PH-Star	TabPFN v1	1	50.0000	22.0000	0.0000	0.0000	0.0000
PH-Star	TabPFN v1	2	38.0000	100.0000	36.0000	2.0000	0.0000
PH-Star	TabPFN v1	3	0.0000	66.0000	91.0000	16.0000	0.0000
PH-Star	TabPFN v1	4	0.0000	10.0000	40.0000	71.0000	13.0000
PH-Star	TabPFN v1	5	0.0000	1.0000	3.0000	12.0000	32.0000

Analysis-version note: earlier PH-Star benchmark fits. The HIOP-GL confusion matrix in Table S10a uses the same earlier benchmark five-seed ensemble as Table S9a, on the original 603-observation temporal-test cohort. It is distinct from the final temporal-validation ensemble summarized in Table S5. Probabilities were averaged across five fits before assigning tiers. The test cohort is the same; the fitted models differ.

Table S10b. Post-development PH-Star confusion matrices for the same five-seed ensembles and $n = 1{,}236$ observations in Table S9b (338 hospitals). Original temporal-test matrices appear in Table S10a ($n = 603$).

Model	True tier	Pred. 1	Pred. 2	Pred. 3	Pred. 4	Pred. 5	Support
HIOP-GL	1	86	46	8	0	0	140
HIOP-GL	2	48	145	147	3	0	343
HIOP-GL	3	7	49	271	58	15	400
HIOP-GL	4	0	0	114	77	77	268
HIOP-GL	5	0	0	5	19	61	85
CatBoost	1	14	104	21	1	0	140
CatBoost	2	3	155	172	13	0	343
CatBoost	3	0	46	292	60	2	400
CatBoost	4	0	0	94	153	21	268
CatBoost	5	0	0	2	40	43	85
Random forest	1	17	99	23	1	0	140
Random forest	2	2	160	178	3	0	343
Random forest	3	0	48	316	36	0	400
Random forest	4	0	0	134	114	20	268
Random forest	5	0	0	8	38	39	85

Rows are observed tiers and columns are predicted tiers. Every matrix totals 1,236; diagonal sums are 640 for HIOP-GL, 657 for CatBoost, and 646 for random forest. These are hospital-release counts, not counts of distinct correctly classified hospitals.

Table S11a. Paired hospital-block QWK comparisons with Holm adjustment.

Target	Comparison	Delta Qwk Bootstrap Mean	Ci Lower	Ci Upper	P Raw	P Holm
PH-Star	HIOP-GL vs ordinal_logistic	0.0453	0.0170	0.0747	<0.002	<0.014
PH-Star	HIOP-GL vs random_forest	0.0092	-0.0289	0.0492	0.6680	0.6680
PH-Star	HIOP-GL vs xgboost	0.0720	0.0303	0.1184	<0.002	<0.014
PH-Star	HIOP-GL vs lightgbm	0.0852	0.0408	0.1338	<0.002	<0.014
PH-Star	HIOP-GL vs catboost	0.0630	0.0204	0.1128	0.0040	0.0120
PH-Star	HIOP-GL vs tabpfn_v1	0.0237	-0.0067	0.0559	0.1540	0.3080
PH-Star	HIOP-GL vs excelformer	0.0889	0.0476	0.1336	<0.002	<0.014

Table S11b. PH-Star five-seed pre-scaling probability-ensemble paired hospital-block intervals for graph, stable-context, head, temporal, view, missingness, and monotonic ablations. Deltas are full minus ablated. PH-VBP intervals are reported separately in Table S11c.

Target	Variant	Metric	Delta Bootstrap Mean	Lower	Upper	P Raw
PH-Star	no_graph	macro_f1	0.0101	-0.0160	0.0357	0.4460
PH-Star	no_graph	qwk	0.0008	-0.0094	0.0117	0.9060
PH-Star	no_graph	mae_t	-0.0118	-0.0364	0.0133	0.4060
PH-Star	no_graph	brier_avg	-0.0023	-0.0035	-0.0011	<0.002
PH-Star	no_graph	low_tier_recall	-0.0429	-0.0850	-0.0061	0.0240
PH-Star	no_stable_node	macro_f1	0.1294	0.0915	0.1687	<0.002
PH-Star	no_stable_node	qwk	0.0757	0.0475	0.1046	<0.002
PH-Star	no_stable_node	mae_t	-0.1984	-0.2517	-0.1443	<0.002
PH-Star	no_stable_node	brier_avg	-0.0182	-0.0219	-0.0144	<0.002
PH-Star	no_stable_node	low_tier_recall	0.0288	-0.0354	0.0866	0.3480
PH-Star	performance_graph_only	macro_f1	0.0621	0.0332	0.0945	<0.002
PH-Star	performance_graph_only	qwk	0.0051	-0.0073	0.0175	0.4140
PH-Star	performance_graph_only	mae_t	-0.0535	-0.0828	-0.0249	<0.002
PH-Star	performance_graph_only	brier_avg	-0.0050	-0.0063	-0.0037	<0.002
PH-Star	performance_graph_only	low_tier_recall	0.0074	-0.0329	0.0487	0.7260
PH-Star	no_stable_node_performance_graph_only	macro_f1	0.1581	0.1196	0.1997	<0.002
PH-Star	no_stable_node_performance_graph_only	qwk	0.0709	0.0452	0.0992	<0.002
PH-Star	no_stable_node_performance_graph_only	mae_t	-0.2103	-0.2625	-0.1606	<0.002
PH-Star	no_stable_node_performance_graph_only	brier_avg	-0.0178	-0.0209	-0.0146	<0.002
PH-Star	no_stable_node_performance_graph_only	low_tier_recall	0.0439	-0.0192	0.0963	0.1440
PH-Star	nominal_head	macro_f1	-0.1343	-0.1758	-0.0932	<0.002
PH-Star	nominal_head	qwk	-0.0196	-0.0433	0.0039	0.0880
PH-Star	nominal_head	mae_t	0.1190	0.0749	0.1636	<0.002
PH-Star	nominal_head	brier_avg	0.0339	0.0248	0.0427	<0.002
PH-Star	nominal_head	low_tier_recall	-0.0945	-0.1489	-0.0358	<0.002
PH-Star	no_temporal	macro_f1	0.0721	0.0396	0.1053	<0.002
PH-Star	no_temporal	qwk	0.0155	0.0014	0.0295	0.0280
PH-Star	no_temporal	mae_t	-0.0782	-0.1093	-0.0447	<0.002
PH-Star	no_temporal	brier_avg	-0.0077	-0.0093	-0.0062	<0.002
PH-Star	no_temporal	low_tier_recall	0.0131	-0.0326	0.0590	0.5580
PH-Star	no_insurance_view	macro_f1	0.0186	-0.0064	0.0460	0.1620
PH-Star	no_insurance_view	qwk	0.0121	-0.0042	0.0289	0.1400
PH-Star	no_insurance_view	mae_t	-0.0351	-0.0662	-0.0050	0.0320
PH-Star	no_insurance_view	brier_avg	-0.0038	-0.0051	-0.0024	<0.002
PH-Star	no_insurance_view	low_tier_recall	-0.0303	-0.0742	0.0128	0.1780
PH-Star	no_safety_proxy	macro_f1	0.0713	0.0374	0.1079	<0.002
PH-Star	no_safety_proxy	qwk	0.0265	0.0121	0.0431	0.0020
PH-Star	no_safety_proxy	mae_t	-0.0897	-0.1296	-0.0532	<0.002
PH-Star	no_safety_proxy	brier_avg	-0.0058	-0.0078	-0.0037	<0.002
PH-Star	no_safety_proxy	low_tier_recall	0.0125	-0.0291	0.0545	0.5460
PH-Star	no_missingness	macro_f1	0.0151	-0.0087	0.0399	0.2280
PH-Star	no_missingness	qwk	0.0032	-0.0076	0.0136	0.5360
PH-Star	no_missingness	mae_t	-0.0164	-0.0400	0.0083	0.2140
PH-Star	no_missingness	brier_avg	-0.0007	-0.0018	0.0003	0.1680
PH-Star	no_missingness	low_tier_recall	-0.0110	-0.0477	0.0277	0.5640
PH-Star	no_monotonic	macro_f1	0.0163	-0.0038	0.0357	0.1200
PH-Star	no_monotonic	qwk	0.0012	-0.0062	0.0080	0.7200
PH-Star	no_monotonic	mae_t	-0.0135	-0.0332	0.0067	0.2060
PH-Star	no_monotonic	brier_avg	-0.0025	-0.0037	-0.0012	<0.002
PH-Star	no_monotonic	low_tier_recall	0.0103	-0.0208	0.0408	0.5280

Table S11c. PH-VBP paired ablation differences on FY2025 tuning data (301 hospitals). Each value compares the selected graph-bypass TPS ensemble with the ablation ensemble.

Ablation	Metric	Difference	Lower 95%	Upper 95%
Adaptive graph, $k = 8$ + TPS	QWK	0.001	-0.014	0.016
	Macro-F1	-0.034	-0.069	-0.002
	MAE_T	0.020	-0.017	0.056
	Low-tier recall	-0.074	-0.143	-0.014
Predefined graph, $k = 5$ + TPS	QWK	0.044	0.016	0.073
	Macro-F1	0.007	-0.046	0.058
	MAE_T	-0.023	-0.080	0.040
	Low-tier recall	0.025	-0.052	0.099
Ordinal head only	QWK	0.124	0.077	0.171
	Macro-F1	0.172	0.114	0.231
	MAE_T	-0.326	-0.429	-0.219
	Low-tier recall	-0.016	-0.109	0.085
No monotonic regularization	QWK	0.010	-0.013	0.034
	Macro-F1	-0.016	-0.055	0.022
	MAE_T	0.003	-0.043	0.047
	Low-tier recall	-0.085	-0.161	-0.008
No healthcare view	QWK	0.035	0.001	0.072
	Macro-F1	-0.008	-0.061	0.042
	MAE_T	-0.027	-0.093	0.037
	Low-tier recall	-0.026	-0.111	0.059
No insurance/cost view	QWK	0.060	0.024	0.101
	Macro-F1	0.024	-0.028	0.080
	MAE_T	-0.070	-0.143	-0.007
	Low-tier recall	0.019	-0.080	0.122
No safety/proxy view	QWK	0.148	0.096	0.205
	Macro-F1	0.091	0.026	0.152
	MAE_T	-0.173	-0.259	-0.090
	Low-tier recall	0.089	-0.023	0.204

Differences are selected minus ablated. Positive values favor the selected model for QWK, Macro-F1 and low-tier recall; negative values favor it for MAE_T. Percentile intervals use 2,000 paired hospital resamples (PCG64 seed 20260909), with the identical draw applied to every ensemble. TPS is averaged across five seeds before applying the FY2024 cutoffs; the ordinal-only ensemble averages probabilities before classification. Low-tier recall averages the separate tier-1 and tier-2 recalls. The original configurations and seeds were rerun to retain individual predictions, reproducing all four seed-level metrics exactly. These descriptive intervals condition on the FY2025 model-selection cohort and fitted ensembles; they are not adjusted for selection or multiplicity. Table S4b separately reports mean ± SD across fits.

Table S12a. Five repeated test-cell masking results for selected methods.

Target	Method	Scenario	Qwk Mean	Qwk Std	Mae T Mean	Mae T Std	Low Tier Recall Mean	Low Tier Recall Std
PH-Star	CatBoost	random_observed_20pct	0.6570	0.0177	0.5038	0.0127	0.3282	0.0098
PH-Star	CatBoost	random_observed_40pct	0.5267	0.0104	0.6126	0.0098	0.2588	0.0079
PH-Star	ExcelFormer	random_observed_20pct	0.6213	0.0240	0.6547	0.0296	0.4287	0.0201
PH-Star	ExcelFormer	random_observed_40pct	0.5310	0.0154	0.7648	0.0116	0.3580	0.0257
PH-Star	HIOP-GL	random_observed_20pct	0.7931	0.0099	0.4660	0.0116	0.5650	0.0308
PH-Star	HIOP-GL	random_observed_40pct	0.7468	0.0085	0.4919	0.0143	0.5391	0.0305
PH-Star	TabPFN v1	random_observed_20pct	0.7374	0.0144	0.5194	0.0134	0.5806	0.0194
PH-Star	TabPFN v1	random_observed_40pct	0.6483	0.0134	0.5910	0.0163	0.4926	0.0267

Table S12b. Corresponding ablated-model module-outage comparisons.

Target	Variant	Comparison	Metric	Delta Bootstrap Mean	Lower	Upper	P Raw
PH-Star	no_healthcare_view	ablated_outage_minus_full_outage	macro_f1	0.1635	0.1025	0.2249	0.0000
PH-Star	no_healthcare_view	ablated_outage_minus_full_outage	qwk	0.2824	0.2143	0.3536	0.0000
PH-Star	no_healthcare_view	ablated_outage_minus_full_outage	mae_t	-0.1500	-0.2297	-0.0731	0.0000
PH-Star	no_healthcare_view	ablated_outage_minus_full_outage	brier_avg	-0.0128	-0.0207	-0.0053	0.0000
PH-Star	no_healthcare_view	ablated_outage_minus_full_outage	low_tier_recall	0.0831	0.0098	0.1614	0.0160
PH-Star	no_insurance_view	ablated_outage_minus_full_outage	macro_f1	-0.0288	-0.0762	0.0199	0.2420
PH-Star	no_insurance_view	ablated_outage_minus_full_outage	qwk	0.0068	-0.0171	0.0298	0.5460
PH-Star	no_insurance_view	ablated_outage_minus_full_outage	mae_t	0.0461	-0.0033	0.0960	0.0600
PH-Star	no_insurance_view	ablated_outage_minus_full_outage	brier_avg	0.0089	0.0042	0.0140	0.0000
PH-Star	no_insurance_view	ablated_outage_minus_full_outage	low_tier_recall	-0.0212	-0.0735	0.0343	0.4600
PH-Star	no_safety_proxy	ablated_outage_minus_full_outage	macro_f1	-0.0349	-0.0791	0.0063	0.0920
PH-Star	no_safety_proxy	ablated_outage_minus_full_outage	qwk	0.0187	-0.0043	0.0440	0.1120
PH-Star	no_safety_proxy	ablated_outage_minus_full_outage	mae_t	0.0257	-0.0182	0.0697	0.2560
PH-Star	no_safety_proxy	ablated_outage_minus_full_outage	brier_avg	0.0010	-0.0031	0.0050	0.6060
PH-Star	no_safety_proxy	ablated_outage_minus_full_outage	low_tier_recall	0.0192	-0.0480	0.0893	0.5860

Table S12c. Five-seed adjacent-tier training-label perturbation results.

Target	Method	Macro F1 Mean	Macro F1 Std	Qwk Mean	Qwk Std	Mae T Mean	Mae T Std	Low Tier Recall Mean	Low Tier Recall Std
PH-Star	CatBoost	0.6007	0.0304	0.7661	0.0140	0.4126	0.0145	0.4209	0.0430
PH-Star	ExcelFormer	0.4803	0.0727	0.6897	0.0339	0.5615	0.0573	0.4311	0.0212
PH-Star	HIOP-GL	0.4239	0.1425	0.7346	0.0847	0.6541	0.1872	0.5272	0.0544
PH-Star	TabPFN v1	0.5918	0.0223	0.7982	0.0162	0.4448	0.0218	0.5853	0.0246

Table S13. Temporal-test HIOP-GL subgroup metrics and sample sizes.

Target	Subgroup Type	Subgroup	Qwk Mean	Qwk Std	Mae T Mean	Mae T Std	Low Tier Recall Mean	Low Tier Recall Std	N Mean	Unique Hospitals Mean
PH-Star	census_region	Midwest	0.7805	0.0139	0.4571	0.0513	0.7167	0.0186	119.0000	60.0000
PH-Star	census_region	Northeast	0.7965	0.0268	0.4944	0.0602	0.4750	0.0693	36.0000	18.0000
PH-Star	census_region	South	0.7904	0.0104	0.4571	0.0382	0.5870	0.0280	322.0000	161.0000
PH-Star	census_region	West	0.8319	0.0136	0.4413	0.0430	0.5204	0.0553	126.0000	63.0000
PH-Star	emergency_group	No	0.6141	0.0233	0.4357	0.0530	0.1538	0.0385	28.0000	15.0000
PH-Star	emergency_group	Yes	0.8165	0.0113	0.4570	0.0399	0.5905	0.0100	575.0000	289.0000
PH-Star	hospital_exposure	Seen in training	0.8238	0.0103	0.4360	0.0385	0.6060	0.0130	572.0000	286.0000
PH-Star	hospital_exposure	Unseen	0.5557	0.0484	0.8258	0.0872	0.3533	0.0477	31.0000	16.0000
PH-Star	ownership_group	Government - Federal	0.7766	0.0262	0.4500	0.0280	0.2500	0.0442	16.0000	8.0000
PH-Star	ownership_group	Government - Hospital District or Authority	0.8256	0.0041	0.4227	0.0267	0.5818	0.0193	308.0000	155.0000
PH-Star	ownership_group	Government - Local	0.7887	0.0214	0.5023	0.0676	0.5951	0.0495	217.0000	109.0000
PH-Star	ownership_group	Government - State	0.8409	0.0252	0.4613	0.0542	0.5375	0.0640	62.0000	31.0000
PH-Star	target_release	2025-02-19	0.8747	0.0127	0.3522	0.0509	0.7023	0.0285	301.0000	301.0000
PH-Star	target_release	2025-04-30	0.7412	0.0155	0.5596	0.0412	0.4698	0.0155	302.0000	302.0000

Table S14. Calibration metrics before and after validation-fitted temperature scaling.

Target	Method	Stage	Brier Avg Mean	Brier Avg Std	Ece 10 Mean	Ece 10 Std	Nll Mean	Nll Std
PH-Star	CatBoost	after_temperature_scaling	0.0954	0.0016	0.1182	0.0095	0.8970	0.0189
PH-Star	CatBoost	before_temperature_scaling	0.0958	0.0010	0.1174	0.0079	0.8746	0.0069
PH-Star	HIOP-GL	after_temperature_scaling	0.1151	0.0044	0.1952	0.0317	1.0621	0.0501
PH-Star	HIOP-GL	before_temperature_scaling	0.1166	0.0033	0.2258	0.0087	1.0752	0.0294
PH-Star	LightGBM	after_temperature_scaling	0.1005	0.0020	0.0990	0.0156	0.9582	0.0288
PH-Star	LightGBM	before_temperature_scaling	0.1022	0.0026	0.1196	0.0334	0.9979	0.0478
PH-Star	Ordinal logistic	after_temperature_scaling	0.1154	0.0000	0.0618	0.0000	1.0116	0.0000
PH-Star	Ordinal logistic	before_temperature_scaling	0.1224	0.0000	0.1495	0.0000	1.1026	0.0000
PH-Star	Random forest	after_temperature_scaling	0.0822	0.0013	0.1050	0.0119	0.8787	0.0135
PH-Star	Random forest	before_temperature_scaling	0.1085	0.0005	0.3186	0.0140	1.0167	0.0045
PH-Star	XGBoost	after_temperature_scaling	0.0981	0.0016	0.0911	0.0122	0.9215	0.0196
PH-Star	XGBoost	before_temperature_scaling	0.0977	0.0015	0.0831	0.0127	0.9151	0.0184
PH-Star	ExcelFormer	after_temperature_scaling	0.1199	0.0055	0.0671	0.0186	1.0720	0.0582
PH-Star	ExcelFormer	before_temperature_scaling	0.1218	0.0061	0.0943	0.0382	1.0956	0.0727
PH-Star	TabPFN v1	after_temperature_scaling	0.1118	0.0021	0.0483	0.0119	0.9833	0.0260
PH-Star	TabPFN v1	before_temperature_scaling	0.1113	0.0020	0.0365	0.0061	0.9766	0.0244

Analysis-version and summary note. Values are means and sample standard deviations across five fits. For HIOP-GL, the calibration analysis uses the earlier benchmark fits underlying Tables S9a and S10a. These are distinct from the final temporal-validation fits used for the HIOP-GL row in Table 3 and the PH-Star summaries in Table S5. The HIOP-GL before-scaling Brier value in this table therefore remains 0.1166 ± 0.0033; the corresponding final temporal-validation five-fit value for Table 3 is 0.1173 ± 0.0027.

Table S15a. Finite-perturbation monotonicity effects and hospital-block intervals.

Target	Feature	N Observations	N Hospitals	Mean Effect Per Sd	Effect Ci Lower	Effect Ci Upper	Violation Rate	Violation Ci Lower	Violation Ci Upper
PH-Star	ALL_MONOTONIC_FEATURES	12100	302	0.0830	0.0780	0.0886	0.0713	0.0631	0.0790
PH-Star	H__TIMELY__ED_2_STRATA_1	97	49	0.0312	0.0177	0.0456	0.2887	0.1808	0.4021
PH-Star	H__TIMELY__ED_2_STRATA_2	48	24	0.0147	0.0056	0.0242	0.2292	0.0833	0.3750
PH-Star	H__TIMELY__OP_18B	598	300	0.0407	0.0353	0.0467	0.1104	0.0803	0.1421
PH-Star	H__TIMELY__OP_18C	524	271	0.0153	0.0103	0.0203	0.3550	0.3080	0.4057
PH-Star	H__TIMELY__OP_22	591	298	0.0566	0.0501	0.0633	0.0745	0.0508	0.1012
PH-Star	H__UNPLANNED__EDAC_30_AMI	308	154	0.0325	0.0277	0.0376	0.0974	0.0552	0.1396
PH-Star	H__UNPLANNED__EDAC_30_HF	599	300	0.1077	0.0985	0.1175	0.0100	0.0017	0.0217
PH-Star	H__UNPLANNED__EDAC_30_PN	595	298	0.1084	0.0986	0.1191	0.0403	0.0218	0.0621
PH-Star	H__UNPLANNED__OP_32	570	287	0.0995	0.0915	0.1081	0.0000	0.0000	0.0000
PH-Star	H__UNPLANNED__OP_35_ADM	385	198	0.0480	0.0424	0.0542	0.0390	0.0182	0.0604
PH-Star	H__UNPLANNED__OP_35_ED	385	198	0.0932	0.0848	0.1025	0.0104	0.0000	0.0234
PH-Star	H__UNPLANNED__OP_36	549	279	0.1066	0.0975	0.1159	0.0128	0.0036	0.0254
PH-Star	H__UNPLANNED__READM_30_AMI	354	177	0.0148	0.0110	0.0187	0.1836	0.1384	0.2288
PH-Star	H__UNPLANNED__READM_30_CABG	178	89	0.0489	0.0401	0.0592	0.0449	0.0112	0.0843
PH-Star	H__UNPLANNED__READM_30_COPD	550	275	0.0785	0.0713	0.0865	0.0364	0.0182	0.0600
PH-Star	H__UNPLANNED__READM_30_HF	599	300	0.0775	0.0707	0.0847	0.0351	0.0183	0.0533
PH-Star	H__UNPLANNED__READM_30_HIP_KNEE	331	166	0.0744	0.0650	0.0850	0.0423	0.0181	0.0725
PH-Star	H__UNPLANNED__READM_30_HOSP_WIDE	603	302	0.0460	0.0406	0.0517	0.0647	0.0448	0.0894
PH-Star	H__UNPLANNED__READM_30_PN	595	298	0.0249	0.0207	0.0287	0.1092	0.0807	0.1426
PH-Star	I__MSPB__MSPB_1	603	302	0.1344	0.1245	0.1455	0.0050	0.0000	0.0116
PH-Star	P__HAI_SIR__HAI_1_SIR	423	213	0.1142	0.0988	0.1301	0.0993	0.0665	0.1348
PH-Star	P__HAI_SIR__HAI_2_SIR	481	242	0.1036	0.0927	0.1154	0.0644	0.0415	0.0898
PH-Star	P__HAI_SIR__HAI_3_SIR	407	210	0.0922	0.0760	0.1105	0.1253	0.0918	0.1597
PH-Star	P__HAI_SIR__HAI_4_SIR	151	78	0.0673	0.0530	0.0847	0.0861	0.0395	0.1448
PH-Star	P__HAI_SIR__HAI_5_SIR	384	192	0.1415	0.1208	0.1629	0.0599	0.0365	0.0885
PH-Star	P__HAI_SIR__HAI_6_SIR	591	297	0.1267	0.1149	0.1398	0.0474	0.0305	0.0660
PH-Star	P__HCAHPS__H_COMP_5_LINEAR_SCORE	601	301	0.1649	0.1470	0.1849	0.0316	0.0167	0.0483

Table S15b. Integrated Gradients absolute attribution by predictor view.

Target	Seed	View	Mean Absolute Ig	Normalized Absolute Ig
PH-Star	20240701	healthcare	0.0095	0.7705
PH-Star	20240701	insurance	0.0011	0.0882
PH-Star	20240701	safety_medication_proxy	0.0017	0.1414
PH-Star	20240702	healthcare	0.0092	0.7956
PH-Star	20240702	insurance	0.0010	0.0835
PH-Star	20240702	safety_medication_proxy	0.0014	0.1209
PH-Star	20240703	healthcare	0.0094	0.7836
PH-Star	20240703	insurance	0.0011	0.0913
PH-Star	20240703	safety_medication_proxy	0.0015	0.1251
PH-Star	20240704	healthcare	0.0082	0.7751
PH-Star	20240704	insurance	0.0009	0.0859
PH-Star	20240704	safety_medication_proxy	0.0015	0.1390
PH-Star	20240705	healthcare	0.0098	0.7721
PH-Star	20240705	insurance	0.0011	0.0889
PH-Star	20240705	safety_medication_proxy	0.0018	0.1389

Supplementary audit notes

The PH-Star performance-similarity relation used 106 continuous predictors. Four rows required the prespecified current-release fallback because the hospital was absent from the immediately preceding release. The FY2024-only PH-VBP selection fit had no within-training annual pair; the FY2024–FY2025 refit contained 284 consecutive same-hospital annual pairs. The nominal-head ablation used ordinary cross-entropy, which does not explicitly encode distance between ordered tiers. Additional-cell masking changed the missingness indicator for every newly masked observed value and did not alter cells already missing. The local PH-Star monotonicity analysis held graph edges fixed and measures model sensitivity in the observed relational context.

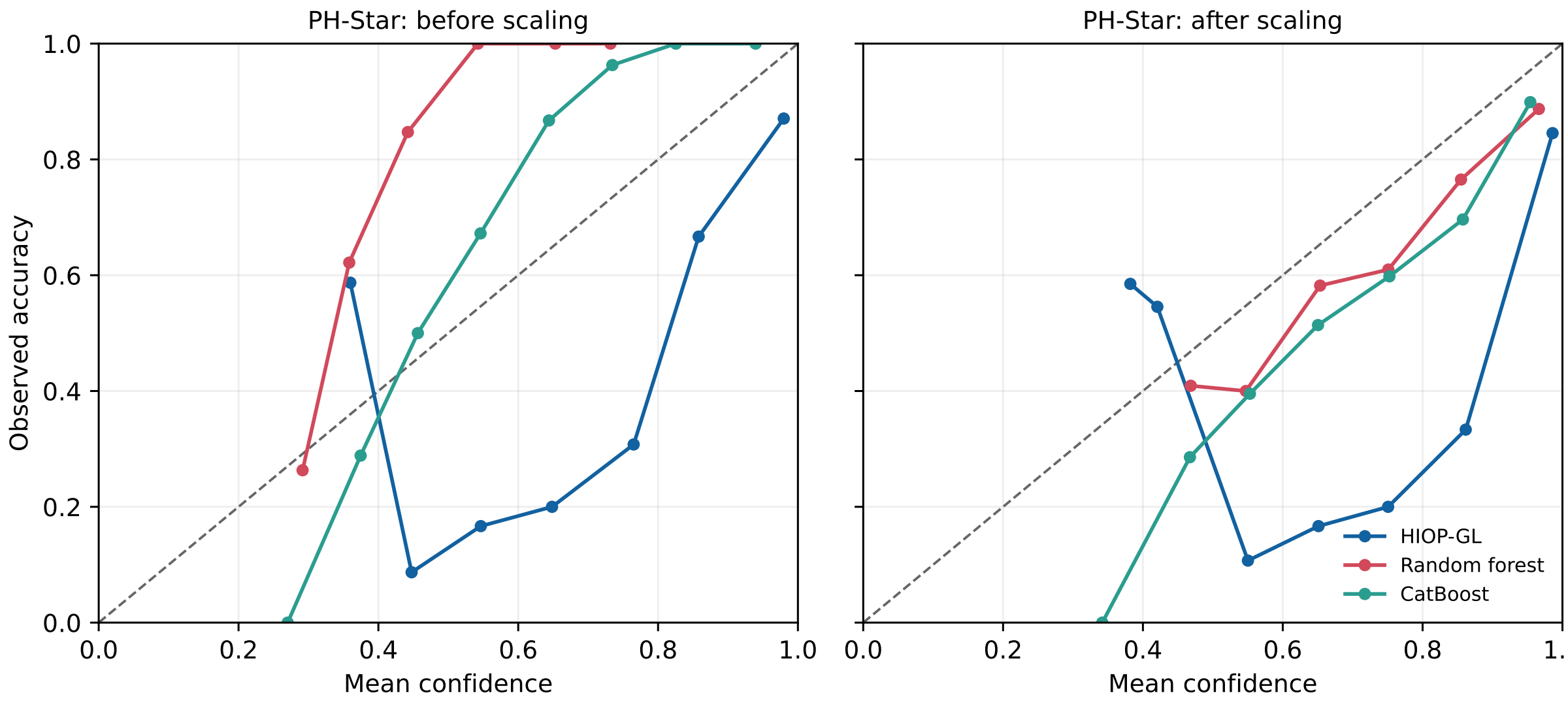

Figure S1: **PH-Star reliability curves before and after validation-fitted temperature scaling.** Curves show confidence-bin accuracy for HIOP-GL, random forest, and CatBoost; empty bins are omitted. The diagonal denotes perfect agreement between mean confidence and observed accuracy. PH-VBP is omitted because the selected pathway predicts continuous TPS followed by deterministic locked cutoffs rather than class probabilities.

Analysis-version and seed note. The curves show the primary seed (20240701) from the archived calibration analysis; Table S14 summarizes all five seeds. The HIOP-GL curve uses the earlier benchmark fit set described in Table S14 and is distinct from the final temporal-validation fits summarized in Table 3 and Table S5.
